# The feasibility of delivering cardiac brief intervention to patients following ST-elevation myocardial infarction: Protocol for a pilot randomised controlled trial

**DOI:** 10.1371/journal.pone.0306406

**Published:** 2024-07-02

**Authors:** Gareth Thompson, Gemma Caughers, Judy Bradley, Patrick Donnelly, Maria Mooney, Donna Fitzsimons

**Affiliations:** 1 School of Nursing and Midwifery, Medical Biology Centre, Queen’s University Belfast, Belfast, United Kingdom; 2 School of Medicine, Dentistry, and Biomedical Sciences, Wellcome Wolfson Institute for Experimental Medicine, Queen’s University Belfast, Belfast, United Kingdom; 3 Ulster Hospital, Cardiovascular Imaging and Research, South Eastern Health and Social Care Trust, Dundonald, United Kingdom; 4 Royal Victoria Hospital, Cardiac Rehabilitation, Belfast Health and Social Care Trust, Belfast, United Kingdom; PLOS: Public Library of Science, UNITED KINGDOM

## Abstract

**Background:**

Patients experience emotional distress and hold cardiac misconceptions following ST-elevation myocardial infarction. These issues informed the co-production of Cardiac Brief Intervention with patients and clinicians. The current study will establish a knowledge base for the feasibility of delivering this intervention to patients following ST-elevation myocardial infarction, with a preliminary exploration of impact on associated outcomes (ClinicalTrials.gov: NCT05848674).

**Methods:**

A pilot randomised controlled trial incorporating a mixed-methods design will be conducted. Patients with ST-elevation myocardial infarction (number = 40) will be recruited from coronary care units at two hospital centres in Northern Ireland, with participants randomised (1:1) to the intervention or control group. Cardiac Brief Intervention constitutes a nurse-led, short (20 minutes) emotional and educational support discussion with a patient, with a leaflet that serves as a memory-aid. It will be delivered to the intervention group prior to discharge from a coronary care unit. The control group will receive standard care information. Data will be collected at baseline, post-intervention, 4 weeks from diagnosis, and 14 weeks from diagnosis. Feasibility measurements and process evaluation (quantitative and qualitative) will assess the viability of the research design and intervention delivery. Cardiac rehabilitation attendance data will be collected, and participants will complete questionnaires related to associated outcomes. Quantitative data will be reported with descriptive statistics and qualitative data will be analysed using framework analysis, with data integrated to achieve triangulation of findings.

**Discussion:**

Educational and emotional difficulties following ST-elevation myocardial infarction may impede patient outcomes and cardiac rehabilitation participation. These issues informed the co-production of Cardiac Brief Intervention with patients and clinicians. This study will evaluate the feasibility of delivering Cardiac Brief Intervention to patients. These results will inform large-scale definitive testing of the intervention, which may lead to adoption in clinical practice to improve cardiac rehabilitation uptake and patient outcomes.

## Introduction

Coronary artery disease (CAD) is characterised by the accumulation of atherosclerotic plaque in the epicardial arteries that perfuse the myocardium [1]. An ST-elevation myocardial infarction (STEMI) represents a potential clinical manifestation of CAD, which comprises a complete blockage of one or more of the coronary arteries, with routine treatment involving an emergency angiography (stent insertion +/- clot removal) [2]. Following reperfusion treatment, patients with STEMI are admitted to a coronary care unit (CCU) to receive specialised care and monitoring from staff [2]. Across the United Kingdom (UK), approximately 100,000 hospital admissions are caused by myocardial infarctions per year, equating to one hospital admission every five minutes [3].

After STEMI, patients are invited to attend cardiac rehabilitation (CR), which is an evidence-based intervention that has been shown to decrease mortality rate, prevent recurrent cardiac events, and enhance quality of life [4]. Despite these clinical benefits, patient uptake to CR programmes in Northern Ireland (NI) is only 49% [5] and generally low worldwide [6], ultimately, resulting in a substantial number of cardiac patients not receiving optimal secondary prevention treatment. In the UK, the importance of maximising patient participation in CR is reflected by the National Health Service Long Term Plan detailing an objective of 85% of eligible patients accessing CR by 2028 [7].

It is well established that patients often hold cardiac misconceptions regarding their myocardial infarction and treatment, which can impede recovery [8]. In addition, patients have reported emotional distress and highlighted a need for more psychological support after their myocardial infarction [9]. Patients and clinicians have informed our research team that a brief personalised intervention prior to discharge may provide an opportunity to correct cardiac misconceptions, provide emotional support, and assist patients with preparation for the next phase of their treatment, including CR [9]. This study will test if the suggested intervention is feasible and conduct a preliminary investigation of impact on associated outcomes.

The research team have acquired considerable knowledge of the needs of local patients in NI through a multi-centre, co-design project that focused on strategies to increase engagement with CR, which involved an exploratory phase of interviews and focus groups with patients and staff involved in CR [10]. The data from this exploratory phase informed a series of co-design workshops attended by patients and staff across the five Health and Social Care Trusts in NI, along with Patient and Public Involvement (PPI) representatives including staff from Northern Ireland Chest, Heart, and Stroke (NICHS) and the British Heart Foundation. Multiple outcomes based on improving engagement with CR were produced. This study relates to one of these outcomes, CArdiac Brief INtervention (CABIN), which constitutes a nurse-led, short (20 minutes) emotional and educational support discussion with a patient, along with a leaflet that serves as a memory-aid.

Across four sessions between September and December 2021, a working group of staff and patients developed the content and wording of CABIN based on their experience of the patient journey, which aligns with Phase 1 of the Medical Research Council (MRC) framework for developing complex interventions [11]. The formatting (*i*.*e*., colour scheme, graphic style, text positioning, and spacing) of the CABIN leaflet was informed by PPI members (patients with CAD). Subsequently, formatting requirements were supplied to Morrow Communications (a Graphic Design Company) to produce the CABIN leaflet. As such, this study will progress CABIN to feasibility testing, in line with Phase 2 of the MRC framework for developing complex interventions [11].

### Aim

To assess the feasibility of delivering CABIN to patients with STEMI prior to discharge from a CCU, with a preliminary exploration of impact on a range of associated outcomes.

### Objectives

1. Evaluate the research design and CABIN delivery through a process evaluation and feasibility measurements, which focus on:
  - Reach, dose, fidelity, data completeness, and acceptability.
  - Context (how external factors influence the delivery and functioning of the study / intervention).
  - Possible mechanisms of impact for the intervention.
2. Preliminary investigation of the impact of CABIN on CR attendance; knowledge of CAD and CR; and psychological and emotional well-being.

## Methods

**Fig 1:**
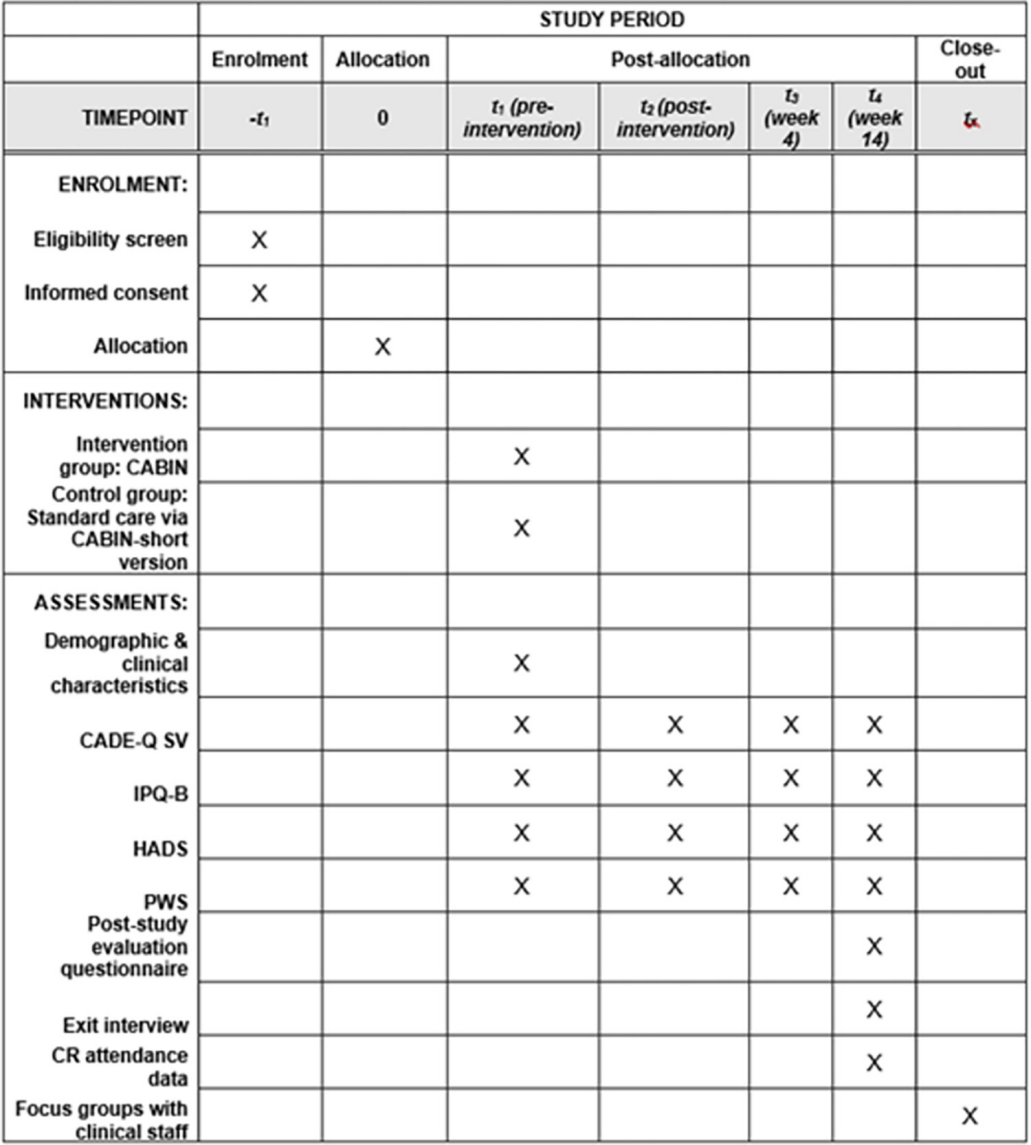
SPIRIT 2013 schedule of enrolment, interventions, and assessments. CABIN = CArdiac Brief INtervention; CADE-Q SV = Coronary Artery Disease Education Questionnaire, Short Version; IPQ-B = Brief Illness Perception Questionnaire; HADS = Hospital Anxiety and Depression Scale; PWS = Personal Wellbeing Score; CR = Cardiac Rehabilitation.

**Fig 2:**
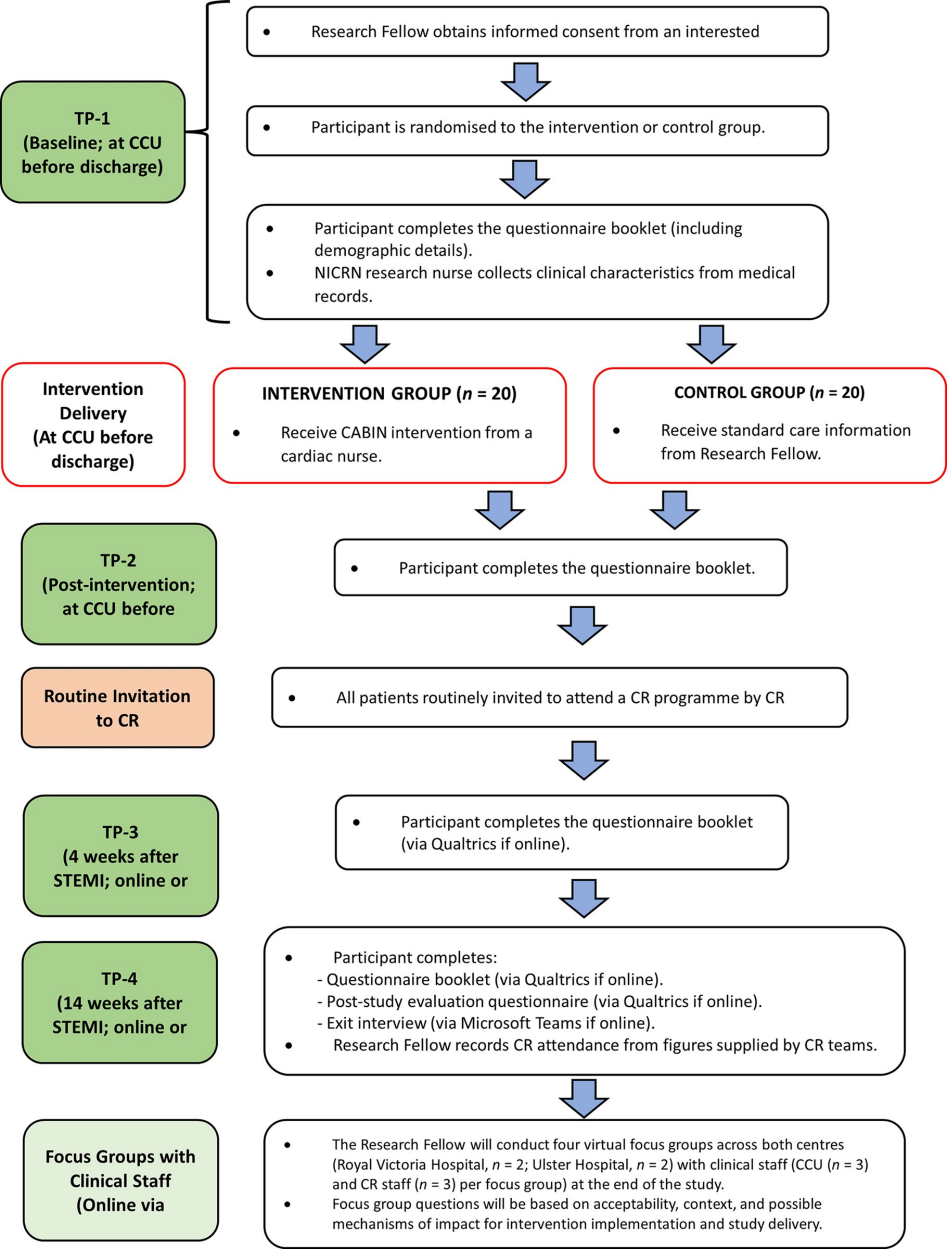
Flow diagram of the study protocol. CCU = Coronary Care Unit; TP = Time Point; NICRN = Northern Ireland Clinical Research Network; CABIN = CArdiac Brief INtervention; STEMI = ST-elevation Myocardial Infarction; CR = Cardiac Rehabilitation; *n* = number.

This study protocol is reported in accordance with the SPIRIT 2013 Statement [12] (Fig 1 and S1 Checklist). This study was registered on ClinicalTrials.gov (ID: NCT05848674). A flow diagram of the study protocol is presented in Fig 2.

### Design

The study will be a pilot randomised controlled trial (RCT) using mixed methods, which aligns with Phase 2 (Feasibility) of the MRC framework for designing complex interventions [11]. This study design was selected as it involves utilising process evaluation and feasibility measurements with the integration of quantitative and qualitative methods to achieve triangulation of findings, which will generate improvements in the feasibility, theory, design, methods, and implementation of CABIN [11, 13]. The study will be guided by the CONSORT extension for reporting pilot and feasibility studies [14].

**PPI input.** This study protocol has been designed in accordance with the National Institute for Health and Care Research INVOLVE guidance [15]. A working group of patients and staff provided the following input on the study protocol:

1. To prevent distress caused by receiving no intervention, the participants of the control group should receive standard care information (details about CAD and stenting / stent placement) in a shortened version of the CABIN leaflet (excluding the supportive discussion with a nurse).
2. The effect of the intervention should be assessed with the Coronary Artery Disease Education Questionnaire, Short Version (CADE-Q SV) [16], which is a validated instrument for evaluating patients’ knowledge of CAD and core components of CR.
3. Implement a distress management protocol in the event of a participant experiencing emotional upset.

A PPI Group has been established to advise on the strategic management / delivery of all project elements (*i*.*e*., commenting on study material, voicing the preferences of patients / public, and providing feedback on study updates / objectives), which will maximise research impact and ensure the interests of patients and public are met. This group comprises patient representatives (4 patients with a history of STEMI) and academic experts. Quarterly meetings will be held via Microsoft Teams or a hybrid approach (Microsoft Teams and in person) depending on preference of members. Communication between meetings will occur as required.

### Theoretical framework

This study will be guided by the ‘Common Sense Model of Self-regulation of Health and Illness’ by Leventhal et al. [17]. This theoretical framework was selected as it facilitates a dynamic understanding of complex behavioural responses to events such as illness [17]. This framework asserts that in response to an illness or health threat, people develop their own common-sense beliefs or illness perceptions about their illness and treatment [17]. These illness perceptions impact the types of healthy behaviours and coping strategies employed by patients for managing their illness, which may influence disease outcomes [17]. This highlights the complex relationship between interpretation of disease and subsequent lifestyle choices, which is applicable to the setting of this study.

### Study setting

Cardiac patients diagnosed with a STEMI will be recruited from two CCUs in NI:

1. Royal Victoria Hospital, Belfast Health and Social Care Trust (BHSCT).
2. Ulster Hospital, South Eastern Heath and Social Care Trust (SEHSCT).

### Sample size

Forty patients diagnosed with a STEMI will be recruited in total (intervention group, number (*n*) = 20; control group, *n* = 20). Whilst a formal sample size is not required for a pilot study [18], the stated sample size was based on a similar study within a cardiac population [19], and aims to balance feasibility of study delivery within available timescale and resources, whilst having a sufficient number of participants to appropriately evaluate feasibility and acceptability. Nonetheless, we will recruit beyond this figure if feasible within time and resources available.

### Recruitment

Approximate figures for 2019 / 2020 indicate that 4,300 patients were admitted to hospitals with a myocardial infarction in NI, with 40% of these patients having a STEMI (*n* = 1,720). 75% of these patients who had STEMI (*n* = 1,290) were treated in the CCUs that will serve as the study centres [20, 21]. Thus, approximately 107 patients with STEMI will be available for recruitment across both study centres per month. Given the small sample size required, we have judged a 6-month period for recruitment to be sufficient (recruiting at least 7% of potential participants per month).

### Recruitment procedure

**Fig 3:**
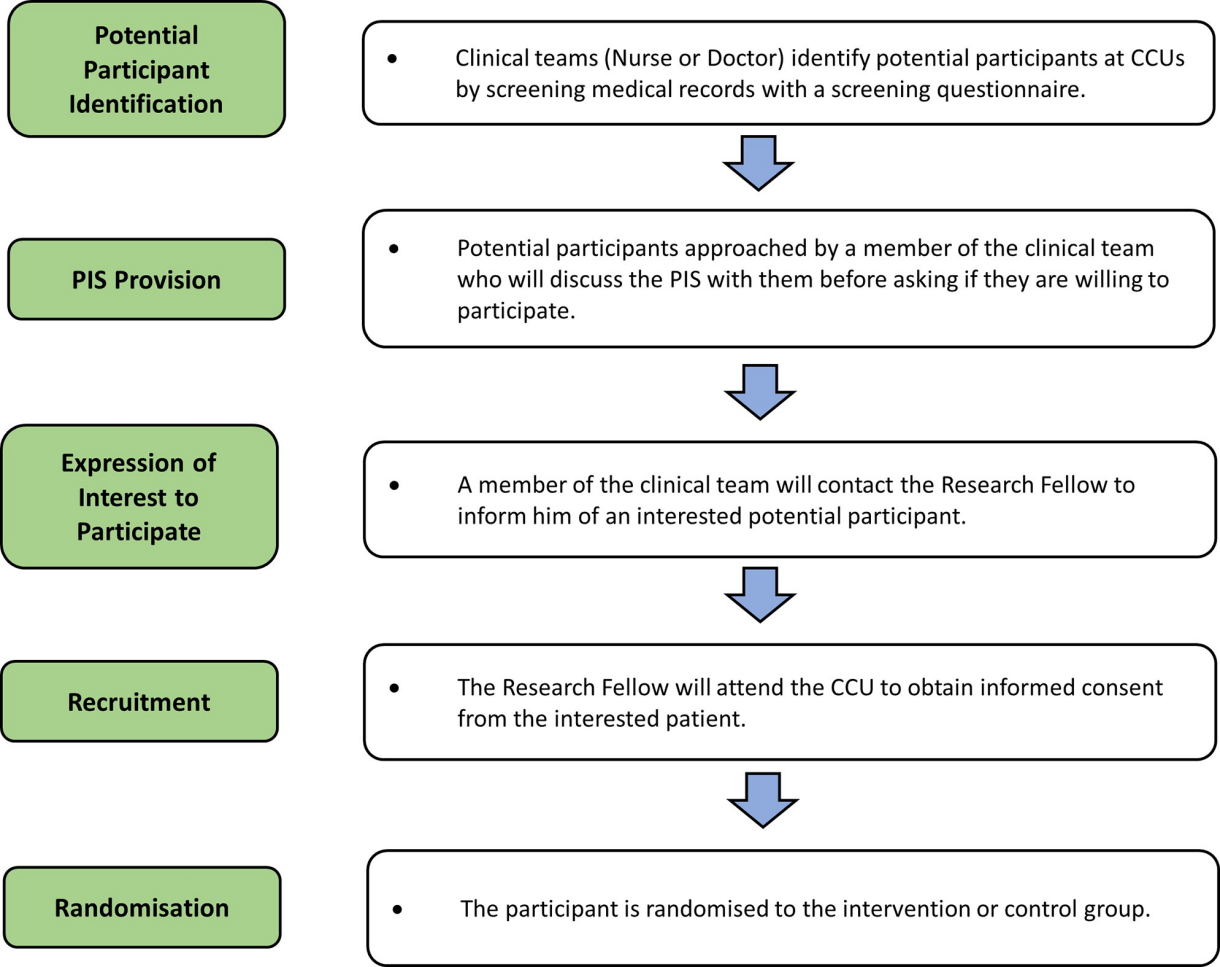
Flow diagram of recruitment procedure. CCUs = Coronary Care Units; PIS = Participant Information Sheets.

The recruitment procedure is demonstrated in Fig 3. A private space will be used for recruitment, such as an empty, private room at the CCU or a quiet area with a privacy screen. Recruited patients will provide written informed consent.

### Randomisation procedure

An online randomisation service (random permuted blocks) provided by Sealed Envelope Ltd [22] will be used by the Research Fellow to randomise (1:1) patients to the intervention or control group. Randomisation will be conducted during the recruitment appointment after informed consent is obtained.

### Eligibility criteria

#### Inclusion criteria

1. Aged 18 years and over.
2. Confirmed diagnosis of STEMI.
3. Physically and mentally capable of participation (judged by Cardiologist or Nurse).
4. Willing to provide informed consent.

#### Exclusion criteria

1. Lacking capacity to give consent (judged by Cardiologist or Nurse).
2. Under the age of 18 years.

#### Study intervention

CABIN will not replace standard care or discharge information routinely delivered to the intervention and control groups by clinical staff.

### CABIN

**Fig 4:**
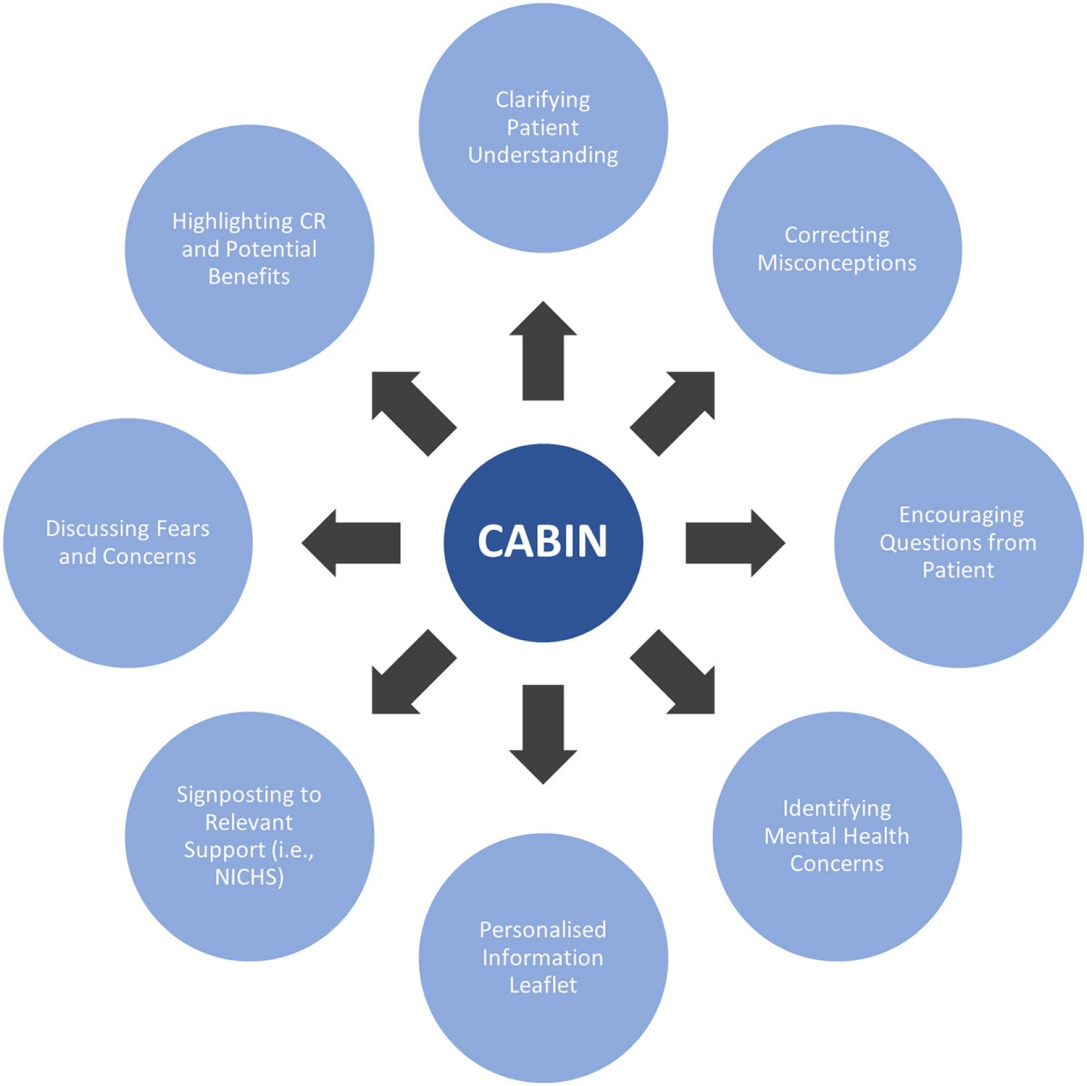
Components of CABIN. CABIN = CArdiac Brief INtervention; NICHS = Northern Ireland Chest, Heart, and Stroke; CR = Cardiac Rehabilitation.

CABIN is designed as a nurse-led, short (20 minutes) emotional and educational support discussion with a patient, with a leaflet that serves as a memory-aid (S1 Fig). The discussion takes place in a quiet area and delivers the eight components in Fig 4 by listening to the patient to identify any concerns he / she may have and correcting erroneous beliefs. CABIN also provides the patient with personalised education on CAD and CR (*i*.*e*., information about stenting, stent placement, medication, and purpose / potential benefits of CR), along with facilitating psychological and emotional support discussions with a nurse (*i*.*e*., explaining causes of a STEMI, discussing support / treatment options, and exploring methods of improving health).

### CABIN delivery

CABIN will be delivered in a single session (one-to-one) to patients of the intervention group before discharge from a CCU. Intervention delivery will be performed by a cardiac nurse with over 20 years of clinical experience in CR. A private space at the CCU will be used for intervention delivery (*i*.*e*., an empty room or a quiet area with a privacy screen). Intervention delivery will take approximately 20 minutes.

### Standard care

The control group will receive standard care information (details about CAD and stenting / stent placement) via a shortened version of the CABIN leaflet (S2 Fig), which excludes psychological and emotional support discussions with a nurse. As per PPI input, this will prevent participants of the control group from feeling disadvantaged by lack of any intervention. This group will enable a comparison of intervention effect to standard care.

### Delivery of standard care

The Research Fellow will deliver (one-to-one) standard care information to patients of the control group prior to discharge from a CCU. A private space at the CCU will be used for delivery (*i*.*e*., an empty room or a quiet area with a privacy screen), with this procedure taking approximately 10 minutes. The Research Fellow will facilitate the delivery of standard care information to the control group to prevent any contamination caused by the cardiac nurse delivering an intervention / information to both groups.

### CR invitation

All patients will be routinely invited to a CR programme by CR teams, which will occur following intervention delivery.

### Data collection

#### Demographic data and clinical characteristics

To set the results in context, demographic details (*i*.*e*., age, gender, education level, and employment status) will be collected from participants via a questionnaire. Informed consent will be requested from participants for the extraction of clinical characteristics (cardiac intervention received, prescribed medication, and co-morbidities) from their medical records. This study will seek adoption by the Northern Ireland Clinical Research Network, which will involve research nurses extracting the required information from medical records of consenting participants.

#### Feasibility measurements and process evaluation

The primary objective of this study is to test the feasibility of the research design and CABIN delivery, which will inform the suitability of methods and procedures for a future effectiveness trial on a larger scale. Thus, the feasibility assessments and process evaluation discussed in the following sections represent processes that are key to the success of a larger study [18].

#### Reach (recruitment rate)

The measurement of recruitment rate will allow the efficiency of the recruitment strategy to be assessed, with this information enabling the identification of potential issues and informing the required duration of the recruitment period for a larger study. Recruitment rate will be reported as the percentage of eligible patients who agreed to participate in the study, and the number of patients recruited monthly per centre over the recruitment period.

#### Dose

Dose will assess the completeness of intervention delivery, which will inform the feasibility of CABIN implementation. This information will be collected via a checklist that is completed by the cardiac nurse following each intervention session.

#### Fidelity

Fidelity will be represented by adherence to the study protocol and procedures, which will identify any modifications required or issues to be resolved. This information will be collected via a study log completed by the Research Fellow, cardiac nurse, and members of the clinical team.

#### Data completeness

The completeness of baseline and outcome measures will be determined as missing data may jeopardise the power of a future study. The Research Fellow will record details about data collection in the study log, which will highlight any problems or required changes to improve data collection for a future study. Data completeness will be reported as the percentage of recruited participants providing data for each baseline and outcome measure, with reasons for any missing data stated.

#### Acceptability, context, and possible mechanisms of impact

A process evaluation of the research design / CABIN delivery will be conducted through measures of:

1. Acceptability of intervention (*i*.*e*., issues for development, required corrections, additional areas for inclusion, aspects enjoyed by participants, and barriers and facilitators to participation).
2. Context (*i*.*e*., factors influencing study / intervention delivery and functioning, for instance, time and resources).
3. Possible mechanisms of impact (*i*.*e*., exploring how intervention activities may trigger change for participants).

These measurements will be primarily assessed with qualitative data collected from exit interviews with participants and focus groups with CCU and CR staff (discussed below). Information from study documents and a post-study evaluation questionnaire will supplement this qualitative data.

#### Protocol for exit interviews with participants

All participants will be eligible to complete a semi-structured interview (approximately 30–60 minutes in duration) at the final Time Point (TP) for data collection. A core set of questions in a “laddered style approach” will be used, which will ensure emerging ideas are explored [23]. Interview questions (S1 Text) have been designed by the research team to focus on acceptability, context, and possible mechanisms of impact for intervention implementation and study delivery. The Research Fellow will conduct each interview individually with participants, which will be held virtually (*i*.*e*., Microsoft Teams) or face-to-face (*i*.*e*., the participant’s home) depending on participant preference. All interviews will be audio-recorded before being transcribed verbatim by an external service. Data analysis of the transcripts will be performed iteratively.

#### Protocol for focus groups with clinical staff

CCU and CR staff at the collaborating hospital centres (Royal Victoria Hospital and Ulster Hospital) will be invited to participate in virtual (*i*.*e*., Microsoft Teams) focus groups (approximately 60 minutes in duration) at the end of the study. A core set of questions in a “laddered style approach” will be utilised to investigate emerging thoughts [23]. These questions (S2 Text) have been created by the research team to focus on acceptability, context, and possible mechanisms of impact for intervention implementation and study delivery. All focus groups will be audio-recorded before being transcribed verbatim by an external service. Data analysis of the transcripts will be performed iteratively.

#### Eligibility criteria

Inclusion Criteria.

1. Aged 18 years and over.
2. Willing to provide informed consent.

Exclusion Criteria.

1. Under the age of 18 years.

#### Sample size

Four virtual focus groups across both centres (Royal Victoria Hospital, *n* = 2; Ulster Hospital, *n* = 2) will be conducted. Each focus group will consist of CCU (*n* = 3) and CR staff (*n* = 3). This sample size is based on recommendations in the literature for a study of this scope [24].

#### Preliminary exploration of CABIN impact

The secondary objective of this study is to perform a preliminary assessment of intervention effect on relevant outcomes, which will inform the selection of appropriate outcome measures for a larger, definitive study.

#### Questionnaires

All participants will be asked to complete four short, validated questionnaires at each TP, which will provide information on the impact of CABIN on knowledge of CAD and CR, and psychological and emotional well-being:

1. CADE-Q SV [16]: evaluates patients’ knowledge of CAD and core components of CR.
2. Brief Illness Perception Questionnaire [25]: rapidly assesses the cognitive and emotional representations of illness.
3. The Hospital Anxiety and Depression Scale [26]: detects states of depression and anxiety.
4. Personal Wellbeing Score [27]: subjectively measures health status and health confidence.

#### CR attendance

The impact of CABIN on CR attendance will be investigated. Participants will be asked to provide informed consent for the Research Fellow to be supplied with their CR attendance figures by the CR teams.

#### Success criteria

A pilot study should possess clearly defined *a priori* success criteria that are based on the outcome measures [18]. These criteria serve as the basis for interpreting the results to ascertain the viability of conducting a larger, definitive study and / or to identify required modifications to promote success [18].

#### Reach (recruitment rate)

Approximate figures for 2019 / 2020 indicate that 1,720 patients were admitted to hospitals with a STEMI in NI [20, 21]. Accordingly, for a future, multi-centre study including patients with STEMI across NI, a sample size of 315 would be required for a 5% margin of error, 95% confidence level, and a response distribution of 50%. Assuming a recruitment period of one year and five centres across the Health and Social Care Trusts in NI, the required monthly recruitment rate per centre would be approximately 6 participants. Therefore, for this study, a monthly recruitment rate of 6 participants per centre will be the success criterion.

#### Data completeness

Missing data for > 20% of participants may pose a serious threat to validity [28]. Therefore, data completeness will be deemed successful if questionnaire data (primary outcome measures of future definitive study) is received from ≥ 80% of participants at each TP.

#### Acceptability of the study protocol and CABIN implementation

Acceptability of the study protocol and CABIN implementation will be predominantly informed by the qualitative data collected from participants and staff. Success will constitute the provision of positive feedback on the study protocol and intervention delivery, along with no infeasible / major revisions being identified.

#### TPs for data collection

Four TPs for data collection were chosen to provide detail of illness perception and emotional / psychological wellbeing for patients between discharge from the CCU and engagement with CR (Fig 2). All timing is from the point of STEMI diagnosis.

### Data analysis

#### Quantitative data analysis

Statistical Package for the Social Sciences (IBM SPSS Statistics, Version 28) will be used for quantitative data analysis. Inferential statistics will not be performed as this study is not formally powered to detect statistical significance. The data will be reported with descriptive statistics. In terms of numerical data, continuous data will be presented as mean ± standard deviation, with discrete data reported as absolute numbers and percentages. Categorical data will be displayed as frequency / percentages. Within and between-group mean differences and 95% confidence intervals will be reported at each TP for the questionnaire data, with results interpreted according to minimum clinically important differences, if available.

#### Qualitative data analysis

Qualitative data will be analysed using the stages of the framework method of analysis; familiarisation, identifying a thematic framework, indexing, charting, mapping, and interpretation [29]. The data in each group will be analysed separately to obtain an account for each experience. The research team will read and re-read transcripts for familiarity, with key issues and themes identified and a thematic framework developed. This framework will be applied to the transcripts and themes will be coded and charted, which will enable the refined themes to be mapped and interpreted to give a full account of the investigated phenomena. The accounts from each group will be compared to find similarities and differences between the groups. Qualitative data analysis will be managed on NVivo (QSR International Pty Ltd. Release 1).

#### Integration of quantitative and qualitative data

The integration of quantitative and qualitative data will follow a triangulation protocol according to the recommendations of Farmer et al. [30]. The findings from the quantitative and qualitative data will be sorted and listed on the same page to ascertain if there is agreement, partial agreement, discrepancy / dissonance, or silence between them. Silence will represent a theme occurring in only one dataset. This assessment will be displayed in a convergence coding matrix, with the triangulated results being discussed by the research team for review and clarification. The reporting of this integrated data will comply with the ‘Good Reporting of a Mixed Methods Study’ framework [31].

#### Ethical considerations

Ethical approval for this study was obtained from the Health and Social Care Research Ethics Committee A (23/NI/0032) in April 2023, with individual governance approvals issued by the BHSCT (22116DF-UC) and SEHSCT (SET.22.38). This study will be conducted in accordance with Good Clinical Practice guidelines and adhere to the Declaration of Helsinki statement (1964). This is a low-risk intervention delivered by an experienced team, with no difficulties anticipated. We have consulted with methodological experts, along with patients and staff in the development of this protocol to mitigate any areas of ethical concern. The intervention was designed by patients and clinical staff according to their experience and expertise.

The identification of eligible patients will be completed by professional gatekeepers (Nurse or Doctor). The Research Fellow will be explicit during the consent process to ensure the patient has fully understood the requirements of the study, which will equip him / her with the knowledge required to provide informed consent. Participants will be informed of their ability to withdraw at any point, without compromising their current clinical care. Medical records will only be assessed by the NICRN research nurses to obtain clinical characteristics if written informed consent is obtained from participants. Study materials have been chosen / created to minimise participant burden. All patient information sheets and informed consent forms are user friendly in design and have been evaluated by PPI representatives. A £20 voucher will be issued to each participant upon study completion as compensation for time commitments. A distress protocol will be implemented in the event of a participant becoming upset. Patients who demonstrate low overall scores on the included questionnaires and scales will be immediately highlighted by the Research Fellow to the healthcare team.

The requirements of the General Data Protection Regulation and Data Protection Act (2018) will be adhered to. All paper data will be stored in locked filing cabinets and all electronic data will be stored on an encrypted, password protected computer on university premises. Only members of the research team will have access to study data. All audio recordings will be destroyed after the transcripts have been checked for accuracy. Personal data will be pseudonymised with a personal identification number system to ensure anonymity. All data presented in publications will be anonymised to protect participant confidentiality.

#### Dissemination

The results of this study will be disseminated via publications in peer-reviewed journals and presented at national and international scientific conferences. Members of the PPI Group will be involved with dissemination of results and co-authorship of papers / abstracts where possible.

#### Rigour

To ensure relevance and acceptability, PPI members informed the selection of validated questionnaires for quantitative data collection. Credibility will be upheld through audio-recording and verbatim transcription of qualitative data, which will enable accurate presentation of participants’ perspectives, using quotations where appropriate [32]. Sensitivity to the qualitative data will be achieved through the research team reading and re-reading transcripts for familiarity, with identified themes being cross-checked by different members of the research team to reduce investigator bias [32]. Member checking will also be implemented to improve trustworthiness, whereby participants are offered an opportunity to assess whether the qualitative analysis accurately reflects their accounts [32].

#### Status and timeline of study

This study has received ethical and governance approvals across the collaborating sites. Recruitment and data collection are currently ongoing and will run from 01/04/2024–01/12/2024. No challenges or changes have occurred.

#### Data availability

All relevant data from this study will be made available upon study completion. Anonymised data sets will be allocated a DOI and transferred to Queen’s University Belfast research information management system (Pure) for long-term preservation, which is publicly accessible and will contain links to the project and relevant publications.

## Discussion

This study seeks to establish an evidence base for delivering additional support at CCUs to patients with STEMI through brief, compassionate discussions between nurses and patients. Iterations of this approach have been tested elsewhere with varying results [33]. However, this project is novel as the intervention has been requested and co-designed by key local stakeholders in NI (*i*.*e*., patients with STEMI and healthcare professionals). If feasible and acceptable, this research will progress to the ‘Evaluation’ phase of the MRC framework for developing complex interventions [11], which will involve performing a definitive, multi-centre RCT to assess the effectiveness of CABIN in a real-world setting. Ultimately, this project could lead to the implementation of CABIN in routine clinical practice at CCUs, which may enhance CR uptake and outcomes for cardiac patients, such as: a reduction in recurrent cardiac events and lower readmission rates for anxiety-related chest pain. In addition, the personalised education delivered by CABIN may empower self-management for patients through improved health literacy.

### Limitations

This is a small-scale, pilot study that will only include two hospital centres in NI, which may decrease the representativeness of the findings. Moreover, only patients with STEMI will be included at this stage of testing to limit confounding variables, such as: differences in illness misperception between patients with STEMI and those with non-STEMI, which can strongly influence the decision to attend CR [34]. Therefore, the results will not have applicability to non-STEMI patients. Finally, effective blinding of participants and staff is not possible due to the obvious nature of the intervention, which may introduce bias to the outcome assessment [35].

## Conclusion

Exploratory work with patients and clinicians in NI identified a need for patients with STEMI to receive a brief, personalised intervention prior to discharge from a CCU. To address this requirement, CABIN was co-designed with patients and clinicians, with the aim of correcting cardiac misconceptions, providing emotional support, and assisting patients with preparation for the next phase of their treatment, including CR. This study will assess the feasibility and acceptability of the research design and intervention delivery, which if successful, will lead to definitive testing of the effectiveness of CABIN in a real-world setting.

## Supporting information

S1 Checklist SPIRIT 2013 checklist. (DOC)

S1 Fig CABIN leaflet. (PDF)

S2 Fig Shortened version of the CABIN leaflet for control group. (PDF)

S1 Text Guide for exit interviews. (DOCX)

S2 Text Guide for focus groups with coronary care unit and cardiac ehabilitation staff. (DOCX)

S3 Text Copy of the protocol that was approved by the ethics committee. (DOCX)

S4 Text Copy of ethical approval. (PDF)

S5 Text Copy of funding approval. (DOCX)
