# Supplementary material for: The feasibility of delivering cardiac brief intervention to patients following ST-elevation myocardial infarction: Protocol for a pilot randomised controlled trial
Source: PLoS One. 2024 Jul 2;19(7):e0306406. doi: 10.1371/journal.pone.0306406 (PMC11218979; doi:10.1371/journal.pone.0306406)
Supplement: S1 Fig — (PDF) [file pone.0306406.s002.pdf]

POSSIBLE CAUSES

What may have caused my heart attack?

This is what the healthcare team can do to help:

Cardiac Rehabilitation | **Medications** | **Cardiologist Follow Up**

These are things **I can do** to help myself:

Notes:

If you have symptoms that persist and are not resolved with GTN spray, call 999

South Eastern Health and Social Care Trust

Belfast Health and Social Care Trust

CABIN

CArdiac Brief INtervention

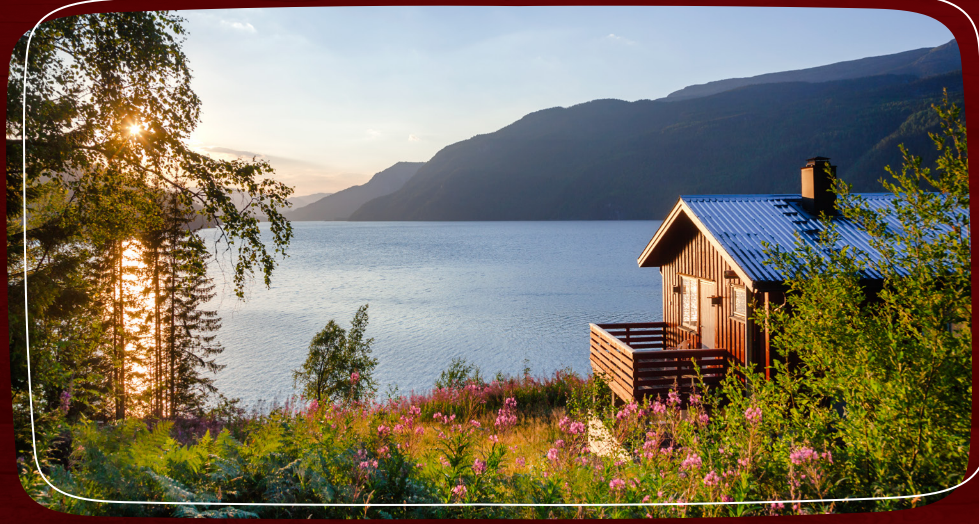

QUEEN'S UNIVERSITY BELFAST

Chest Heart & Stroke

CArdiac Brief INtervention

We know you have had a heart attack

Sometimes you just need a quiet space to talk things over before you make any decisions

CABIN has been designed with patients and nurses to help you get your head around what has happened.

We will give you a chance to discuss ways to prevent future heart attacks and you can talk to a healthcare professional about how to find your way to a healthy future.

What happened to your heart?

Imagine your heart as the size of a fist. It is a pump carrying blood to the body.

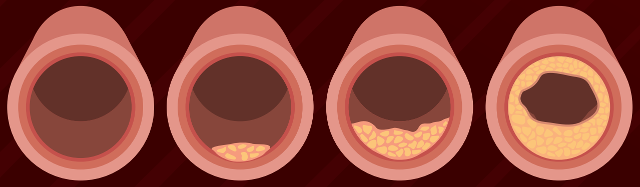

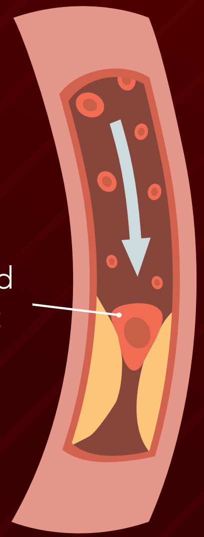

The small vessels (size of pencil lead) on the surface carry blood to the heart.

These vessels can become narrowed or blocked, causing a heart attack.

YOUR HEART

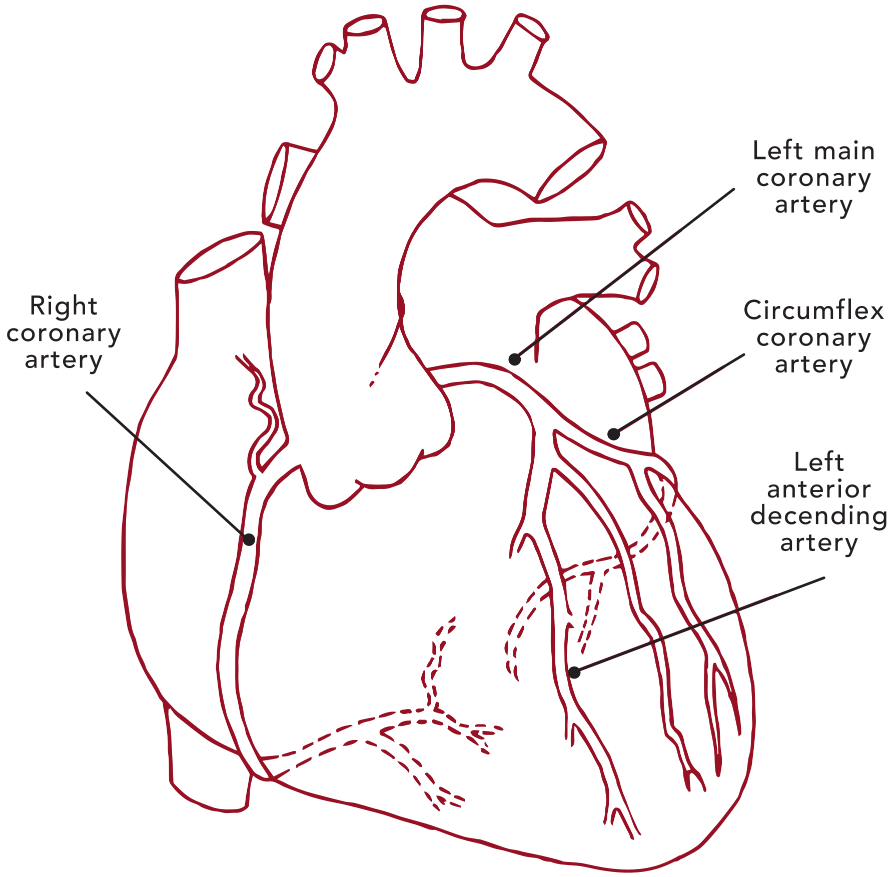

Shaded areas show where the vessels are narrow

X marks position of your stent

Coronary Heart Disease is a lifelong illness

Stenting is a very good treatment - but it is not a cure
