# Supplementary material for: The feasibility of delivering cardiac brief intervention to patients following ST-elevation myocardial infarction: Protocol for a pilot randomised controlled trial
Source: PLoS One. 2024 Jul 2;19(7):e0306406. doi: 10.1371/journal.pone.0306406 (PMC11218979; doi:10.1371/journal.pone.0306406)
Supplement: S2 Fig — (PDF) [file pone.0306406.s003.pdf]

South Eastern Health and Social Care Trust

Belfast Health and Social Care Trust

CABIN

CArdiac Brief INtervention

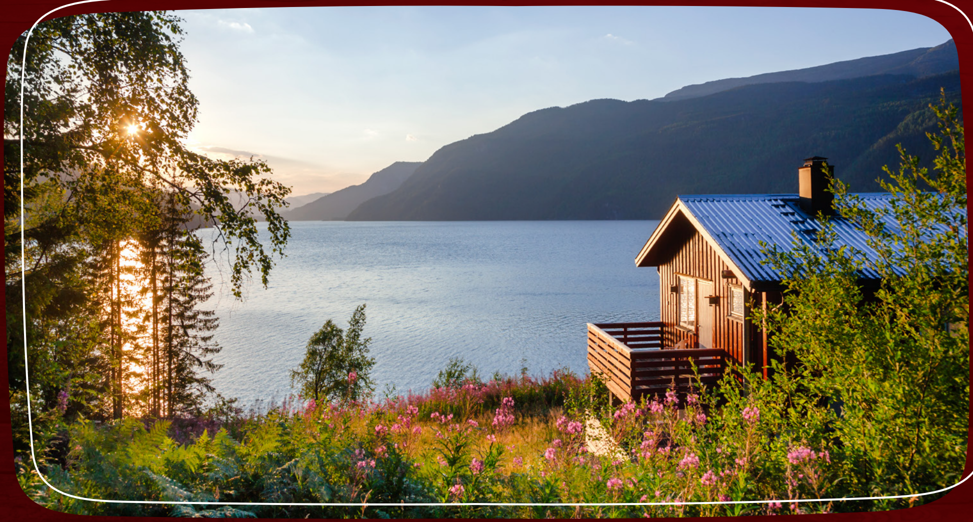

QUEEN'S UNIVERSITY BELFAST

Chest Heart & Stroke

What happened to your heart?

Imagine your heart as the size of a fist.
It is a pump carrying blood to the body.

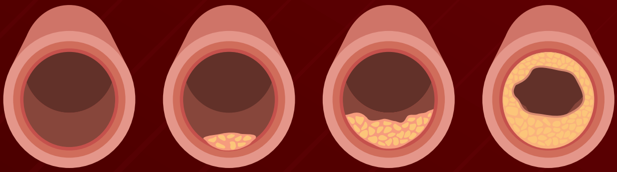

The small vessels (size of pencil lead) on the surface carry blood to the heart.

These vessels can become narrowed or blocked, causing a heart attack.

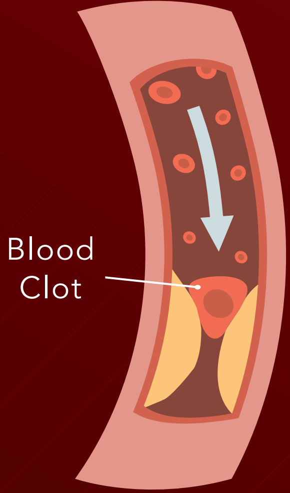

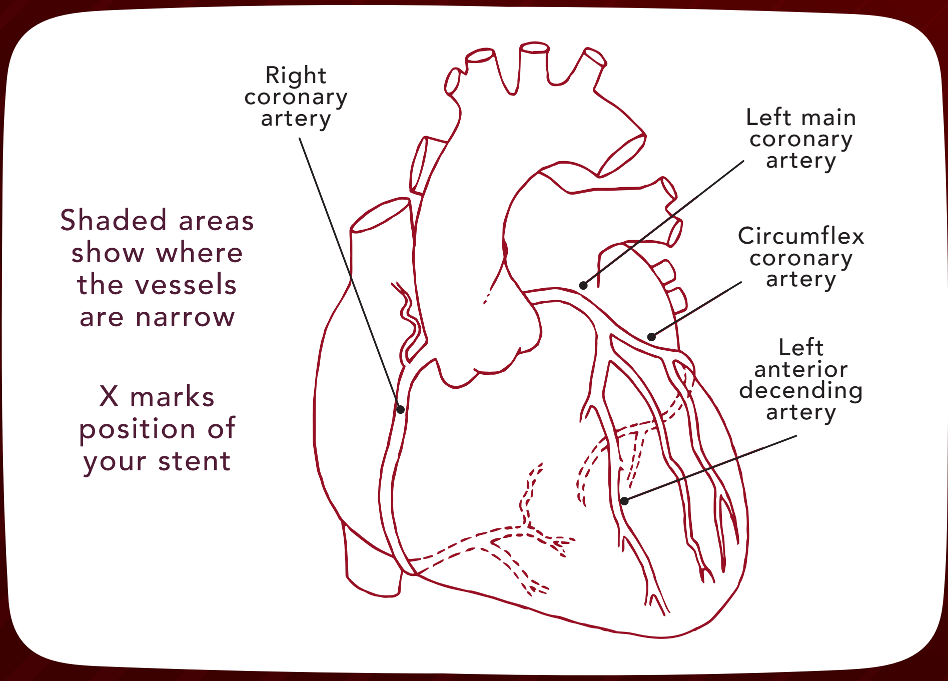

If you have symptoms that persist and are not resolved with GTN spray, call 999
