# Supplementary material for: The feasibility of delivering cardiac brief intervention to patients following ST-elevation myocardial infarction: Protocol for a pilot randomised controlled trial
Source: PLoS One. 2024 Jul 2;19(7):e0306406. doi: 10.1371/journal.pone.0306406 (PMC11218979; doi:10.1371/journal.pone.0306406)
Supplement: S1 Text — (DOCX) [file pone.0306406.s004.docx]

**S3 Text. Guide for exit interviews.**

**Intervention group participants:**

**Broad invitation statement**

1) Can you tell me how you felt after your heart attack (*i.e.,* physically, emotionally, and socially)?

**Follow-up topics**

2) Can you talk about your experience with receiving the CABIN intervention?

3) Did you enjoy / dislike any aspects of CABIN?

4) Is there anything about the CABIN intervention you think we should change or do differently?

5) Can you think of anything that we could do to help patients get the CABIN intervention whilst in hospital after a heart attack?

6) Can you think of anything that would stop us from giving CABIN to patients whilst they are in hospital after a heart attack?

7) How did receiving the CABIN intervention impact you (*i.e.,* triggering change, emotional support, and cardiac rehabilitation attendance)?

8) How did you feel as a participant in this study?

9) Is there anything about the study you think we should change or do differently (*i.e.,* outcome measures and time points for data collection)?

10) Can you think of anything that would help patients take part in a study like this?

11) Can you think of anything that would stop patients from taking part in a study like this?

**Control group participants:**

**Broad invitation statement**

1) Can you tell me how you felt after your heart attack (*i.e.,* physically, emotionally, and socially)?

**Follow-up topics**

2) Can you talk about your experience as a participant in this study?

3) Did you enjoy / dislike any aspects of this study?

4) Is there anything about the study you think we should change or do differently (*i.e.,* outcome measures and time points for data collection)?

5) Can you think of anything that would help patients take part in a study like this?

6) Can you think of anything that would stop patients from taking part in a study like this?

7) How did receiving the shortened version of CABIN impact you?

8) Is there anything about the shortened version of CABIN you think we should change or do differently?

9) Can you think of anything that we could do to help patients get the full CABIN intervention whilst in hospital after a heart attack?

10) Can you think of anything that would stop us from giving the full CABIN intervention to patients whilst they are in hospital after a heart attack?
