# Supplementary material for: The feasibility of delivering cardiac brief intervention to patients following ST-elevation myocardial infarction: Protocol for a pilot randomised controlled trial
Source: PLoS One. 2024 Jul 2;19(7):e0306406. doi: 10.1371/journal.pone.0306406 (PMC11218979; doi:10.1371/journal.pone.0306406)
Supplement: S2 Text — (DOCX) [file pone.0306406.s005.docx]

**S4 Text.** **Guide for focus groups with coronary care unit and cardiac rehabilitation staff.**

**Broad invitation statement**

1) How does everyone feel about the CABIN study (*i.e.,* study rationale and potential impact)?

**Follow-up topics**

2) What are the strengths and limitations of the CABIN intervention?

3) Is there anything about the CABIN intervention we should change or do differently?

4) Is there anything we could do to help patients get the CABIN intervention whilst in hospital after a STEMI?

5) Is there anything that would stop us from giving CABIN to patients whilst they are in hospital after a STEMI (*i.e.,* time and resources)?

6) How would CABIN impact patients after a STEMI (*i.e.,* triggering change, emotional support, and cardiac rehabilitation attendance)?

7) Is there anything about the study we should change or do differently (*i.e.,* recruitment strategy, intervention delivery, outcome measures, and time points for data collection)?

8) Is there anything that would help us perform a study like this (*i.e.,* recruitment, intervention delivery, and data collection)?

9) Is there anything that would stop us from performing a study like this?
