# Supplementary material for: The feasibility of delivering cardiac brief intervention to patients following ST-elevation myocardial infarction: Protocol for a pilot randomised controlled trial
Source: PLoS One. 2024 Jul 2;19(7):e0306406. doi: 10.1371/journal.pone.0306406 (PMC11218979; doi:10.1371/journal.pone.0306406)
Supplement: S3 Text — (DOCX) [file pone.0306406.s006.docx]

**CABIN – (CArdiac Brief INtervention): a feasibility study to promote engagement with cardiac rehabilitation through an early, personalised, and holistic intervention.**

# **Study Title**

CABIN – (CArdiac Brief INtervention): a feasibility study to promote engagement with cardiac rehabilitation through an early, personalised, and holistic intervention.

# **Contact**

**Name:** Dr Gareth Thompson

**Post:** Research Fellow

**Address:** Room No. 05.310, School of Nursing Midwifery, Queen’s University Belfast, Medical Biology Centre, 97 Lisburn Road, Belfast, BT9 7BL.

**Tel:** 02890972696 (work), 07714198795 (mobile)

**Email:** [gareth.thompson@qub.ac.uk](mailto:gareth.thompson@qub.ac.uk)

# **Research Team**

Professor Donna Fitzsimons (Principal Investigator), Head of School of Nursing and Midwifery, Queen’s University Belfast.

Professor Judy Bradley (Alternative Principal Investigator), Director of Clinical Research Facility, School of Medicine, Dentistry, and Biomedical Sciences, Queen’s University Belfast.

Dr Gareth Thompson (Research Fellow), School of Nursing and Midwifery, Queen’s University Belfast.

Gemma Caughers (Alternative Research Fellow), Lecturer (Education), School of Nursing and Midwifery, Queen’s University Belfast.

Dr Patrick Donnelly (Clinical Contact), Consultant Cardiologist, Ulster Hospital, South Eastern Health and Social Care Trust.

Dr Clare McKeaveney (Expert Advisor – Statistics), Lecturer, School of Nursing and Midwifery, Queen’s University Belfast.

Professor Mike Clarke (Expert Advisor – Research Methodology), Director of MRC Methodology Hub, School of Medicine, Dentistry, and Biomedical Sciences, Queen’s University Belfast.

Maria Mooney (Research Assistant), Royal Victoria Hospital, Belfast Health and Social Care Trust.

# **Abstract**

## **Background**

An ST-elevation myocardial infarction (STEMI) is a specific type of heart attack, following which cardiac rehabilitation (CR) is indicated to save lives and prevent recurrence. Patients often struggle to comprehend the cardiac event and hold erroneous beliefs regarding STEMI. These issues can impede recovery, including willingness to attend CR, with less than 50% uptake rates locally. Previous research identified the need for more psychological support after STEMI, which informed the co-production of a relevant intervention (CABIN – CArdiac Brief INtervention) with patients and clinicians.

## **Aim**

To assess the feasibility of delivering CABIN and explore associated outcomes following STEMI.

## **Methods**

A mixed-methods study based on the Medical Research Council framework for developing complex interventions will be conducted. Patients who had STEMI (number = 40) from 2 hospital centres in Northern Ireland will be recruited to a feasibility randomised controlled trial. CABIN will constitute a brief leaflet that provides personalised education on coronary artery disease (CAD) and CR, along with facilitating psychological and emotional support discussions with a nurse. It will be delivered to the intervention group prior to discharge from a Coronary Care Unit. Data collection will be conducted across both groups (intervention and control) at baseline, post-intervention, 3-4 weeks from diagnosis, and 14 weeks from diagnosis (in line with the start and end of CR). Feasibility measurements and process evaluation (quantitative and qualitative) will assess the viability of the research design and CABIN delivery. Attendance data will be collected to examine participant engagement with CR. Participants will complete questionnaires to evaluate intervention effect on psychological and emotional well-being, along with knowledge of CAD and CR. Quantitative data will be reported with descriptive statistics and qualitative data will be analysed using framework analysis.

## **Anticipated Impact**

If feasible and acceptable, effectiveness of CABIN will be assessed in a multi-centre trial. This intervention could lead to greater CR uptake and improved outcomes for cardiac patients including prevention of recurrent cardiac events, reduced anxiety, improved health literacy, and better engagement with local support networks.

# **Study Protocol**

## **Background**

This project seeks to develop and test an innovative intervention for patients who have suffered a specific type of myocardial infarction / heart attack called an ST-elevation myocardial infarction (STEMI) and have subsequently been referred to cardiac rehabilitation (CR). There are two types of heart attack: 1) STEMI and 2) Non-ST elevation myocardial infarction (NSTEMI). A STEMI occurs secondary to a complete blockage of one or more of the coronary arteries responsible for supplying the myocardium with blood and oxygen, with treatment involving a visit to a Coronary Catheterisation Laboratory for an emergency angiography (stent insertion +/- clot removal). After STEMI, patients are invited to attend CR following discharge from hospital. Importantly, these patients often perceive their condition as serious and life threatening (1). The project will only include patients who had STEMI at this stage of testing to remove confounding variables, such as: illness misperception, which can strongly influence decision to attend CR (1–3).

CR is an evidence-based intervention delivered after cardiac patients have been discharged, which has proven to be successful in decreasing mortality rate, preventing recurrent cardiac events, and enhancing quality of life (4). Along with the risk reduction and physical benefits offered by CR, there are psychological advantages, with completing the programme potentially improving quality of life (5). Over the past two decades, CR has been delivered using a variety of methods, for example, face to face, digital, telephone and self-directed study (6–9). Despite the wide range of delivery options available, patient engagement with CR programmes remains low and in Northern Ireland (NI) only 49% of eligible patients will take their place in CR (10). This is a global problem impacting Western and developed nations alike and remains a priority for the World Heart Foundation (11). Despite the benefits of completing a CR programme, there are still many reasons for patients not to engage, including: family responsibilities, work commitments, social issues, and logistical challenges (12–14). It is well established that patients often hold erroneous beliefs, known as cardiac misconceptions, regarding their heart attack and treatment, which can impede recovery (15), with psychological factors also strongly influencing their decisions (16,17). In a 2015 study by members of the research team (GC, PD, and DF), patients who experienced STEMI or NSTEMI discussed fear, guilt, anxiety, and low mood during the first days following their heart attack (2). More recently, local patients have highlighted a need for more psychological support after their heart attack (18). These findings are extensively corroborated in the literature, with patients being physically and emotionally shaken by their experience; they are shocked and afraid and often just want to go home and put it behind them (19). Importantly, patients, staff, and Northern Ireland Chest, Heart, and Stroke (NICHS) representatives informed us that a brief personalised intervention prior to discharge may provide the opportunity to correct misconceptions, emotionally support the patient, and help patients prepare for the next phase of their treatment, including CR (18). This study will test if the suggested intervention is feasible and explore associated outcomes.

Within standard practice, the multidisciplinary team focus on a safe patient discharge from the ward; discussing diagnosis and medication; and directing patients towards the CR programme. In some NI hospitals, CR nurses can attend Coronary Care Units (CCUs) to inform patients about CR and discuss practical concepts, such as: risk factors and lifestyle changes for the period between discharge and commencing the core CR programme. Factors including fast patient turnover, weekend discharges, staff shortages, and more recently, COVID-19 restrictions, all impact the ability of CR nurses to undertake this first meeting. In addition to these matters, time constraints result in limited opportunities to discuss the emotional and psychological impact on the patient and their family. Patients report that they are not psychologically ready to start this stage of their recovery, as they are still processing what has happened to them (12). CCU Nurses are ideally placed and suitably qualified to provide additional support to patients through brief, reassuring, and compassionate discussions. This study seeks to formalise this discussion process by facilitating consistent, personalised information for patients with STEMI, and providing them with access to essential psychological and emotional support. Iterations of this approach have been tested elsewhere with varying results (19). However, this project is novel as the intervention has been identified as necessary by local patients and subsequently co-designed by staff and patients. Thus, CABIN aims to formalise psychological and emotional support discussions using a personalised approach and provides a safe space for patients to discuss concerns.

### **Development of CABIN**

The research team have acquired considerable knowledge of local patients’ needs through a Public Health Agency funded doctoral research fellowship study (GC), which focused on strategies to increase engagement with CR and was a multi-centre, co-design project with an exploratory phase of interviewing and focus groups with patients and staff involved in CR (12). The data from this exploratory phase informed a series of co-design workshops attended by previous participants and Patient and Public Involvement (PPI) representatives including NICHS and British Heart Foundation staff. Participants contributed from each of the five Health and Social Care Trusts. Multiple outcomes that focused on improving engagement with CR were produced. This study relates to one of these outcomes (CABIN), which constitutes a brief leaflet that provides patients with personalised education on coronary artery disease (CAD) and CR, along with psychological and emotional support discussions facilitated by a nurse (18). Across four sessions between September and December 2021, a working group of staff and patients developed the content and wording of CABIN based on their experience of the patient journey, which aligns with Phase 1 of the Medical Research Council (MRC) framework for developing complex interventions (20). The working group also provided the following input:

1) To minimise bias, participants of the control group should receive a refined version of CABIN (excluding supportive discussion), which details some standard discharge information (information about CAD and stenting / stent placement).

2) The immediate effect of the intervention should be assessed with the Coronary Artery Disease Education Questionnaire, Short Version (CADE-Q SV) (21), which is a validated instrument for evaluating patients’ knowledge of CAD and core components of CR.

3) Implement a distress management protocol in the event of a participant experiencing emotional upset.

The formatting (*i.e.,* colour scheme, graphic style, text positioning, and spacing) of CABIN was informed by PPI members (patients with CAD) from the Cardiac Research Group within the School of Nursing and Midwifery at Queen’s University Belfast (QUB). Subsequently, formatting requirements were supplied to Morrow Communications (a Graphic Design Company) to produce CABIN. As such, funding has been secured from NICHS for this study to progress CABIN to feasibility testing, in line with Phase 2 of the MRC framework for developing complex interventions (20).

## **Research Question**

Is the delivery of CABIN to patients with STEMI, prior to discharge, feasible, and does the intervention increase engagement with CR and improve psychological and emotional well-being in comparison to standard care?

### **Aim**

To assess the feasibility of delivering CABIN to patients with STEMI, prior to discharge, and to explore a range of associated outcomes.

### **Objectives**

1) Evaluate the research design and CABIN implementation through a process evaluation and feasibility measurements, which focus on:

1. Reach, dose, fidelity, data completeness, and acceptability.
2. Context (how external factors influence the delivery and functioning of the study / intervention).
3. Possible mechanisms of impact for the intervention.

2) Preliminary exploration of the impact of CABIN on CR attendance; knowledge of CAD and CR; and psychological and emotional well-being.

## **Methods**

The study is based on the MRC framework for designing complex interventions (20) and implements a mixed-methods design. This will involve utilising process evaluation and feasibility measurements that integrate quantitative and qualitative methods to achieve triangulation of findings and generate improvements in the feasibility, theory, design, methods, and implementation of CABIN (20,22).

### **Theoretical Framework**

This study will be guided by the ‘Common Sense Model of Self-regulation of Health and Illness’ by Leventhal et al. (23). This theoretical framework was selected as it facilitates a dynamic understanding of complex behavioural responses to events such as illness (23). This framework asserts that in response to an illness or health threat, people develop their own common-sense beliefs or illness perceptions about their illness and treatment (23). These illness perceptions impact the types of healthy behaviours and coping strategies employed by patients for managing their illness, which may influence disease outcomes (23). This highlights the complex relationship between interpretation of disease and subsequent lifestyle choices, which is applicable to the setting of this study.

### **Study Design**

CABIN will be assessed with a feasibility randomised controlled trial (RCT). The study will be guided by the CONSORT extension for reporting pilot and feasibility studies (24). Ensuring patient inclusion and preference is a priority for the research team, therefore, this study protocol has been designed in accordance with NIHR INVOLVE guidance (25).

##### **PPI Group**

A PPI Group has been established to advise on the strategic management / delivery of all project elements (*i.e.,* commenting on study material, voicing the preferences of patients / public, and providing feedback on study updates / objectives), which will maximise research impact and ensure the interests of patients / public are met. This group comprises patient representatives (4 patients with history of STEMI) and academic experts. Quarterly meetings will be held via Microsoft Teams or a hybrid approach (Microsoft Teams and in person) depending on preference of members. Communication between meetings will occur as required.

##### **Operational Research Team**

The Operational Research Team (DF, JB, GT, and GC) will cooperate to provide the Research Fellow (GT) with oversight and guidance for the daily management and delivery of CABIN. Monthly meetings will be held via Microsoft Teams or a hybrid approach (Microsoft Teams and in person) depending on preference of members. Communication between meetings will occur as required.

##### **General Research Team**

The General Research Team (DF, JB, GT, GC, PD, CMcK, MC, and MM) will oversee project management / delivery, research methodology, and procedures for CABIN (*i.e.,* discussing and agreeing on protocols, research methodology, and procedures for implementation). Meetings will be held every two months via Microsoft Teams or a hybrid approach (Microsoft Teams and in person) depending on preference of members. Communication between meetings will occur as required.

#### **Setting**

Two CCUs in NI:

1) Royal Victoria Hospital, Belfast Health and Social Care Trust (BHSCT).

2) Ulster Hospital, South Eastern Heath and Social Care Trust (SEHSCT).

#### **Population**

Cardiac patients diagnosed with a STEMI and admitted to a CCU.

##### **Inclusion criteria**

1) Aged 18 and over.

2) Confirmed diagnosis of STEMI.

3) Physically and mentally capable of participation (judged by Cardiologist or Nurse).

4) Willing to provide informed consent.

##### **Exclusion criteria**

1) Lacking capacity to give consent (judged by Cardiologist or Nurse).

2) Under the age of 18.

#### **Intervention Design**

There are eight components that form the basis of CABIN, as shown in Figure 1.


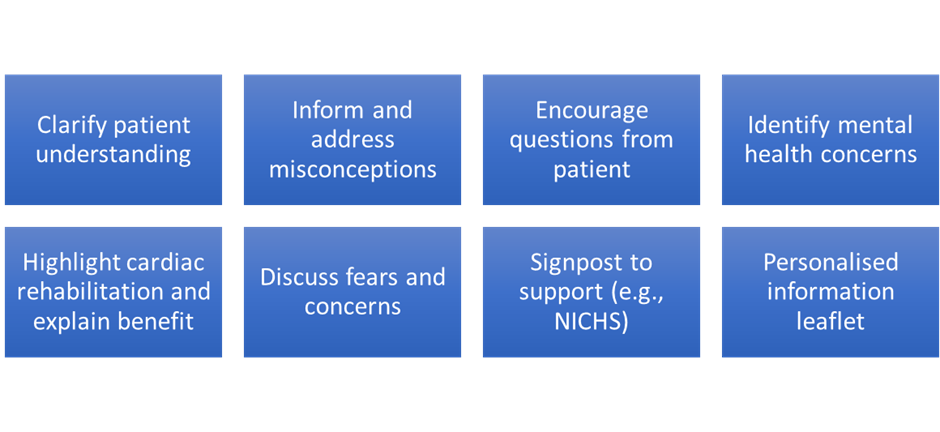


*Figure 1.* Components of CABIN.

CABIN is designed as a brief intervention with a facilitated discussion between a patient and a nurse, with the provision of a short leaflet (see Appendix 1) that summarises information and acts as an aide memoire. The discussion takes place in a quiet area and is intended to last 15-20 minutes. It addresses the eight components in Figure 1 by listening to the patient and recognising any concerns he / she may have or identifying erroneous beliefs. It also provides the patient with personalised education on CAD and CR (*i.e.,* information about stenting, stent placement, medication, and purpose / potential benefits of CR), along with facilitating psychological and emotional support discussions with a nurse (*i.e.,* explaining causes of a STEMI, discussing support / treatment options, and exploring methods of improving health).

#### **Comparator**

##### **Control Group**

In collaboration with our working group, we have considered the needs of the control group and agreed that a short educational session should be conducted to minimise bias and achieve ethical best practice. The control group will receive a refined version of CABIN (see Appendix 2), which excludes psychological and emotional support discussions with a nurse and details some standard discharge information (information about CAD and stenting / stent placement). This will prevent participants of the control group from feeling disadvantaged by lack of any intervention.

Both forms of CABIN will not replace standard care or discharge information routinely delivered by clinical staff.

#### **Sample Size**

40 patients diagnosed with a STEMI will be recruited in total (intervention group, number (*n*) = 20; control group, *n* = 20). Whilst a formal sample size is not required for a feasibility study (26), the stated sample size was based on similar studies within a cardiac population (27,28), and aims to balance feasibility of study delivery within available timescale and resources, whilst having a sufficient number of participants to appropriately evaluate feasibility and acceptability. Nonetheless, we will recruit beyond this figure if feasible within time and resources available.

#### **Recruitment**

The study will comply with all regulatory requirements. Prior to study initiation, ethical and governance approvals will be sought from QUB, National Health Service (NHS) / Health and Social Care (HSC) Research Ethics Committee (REC), and local HSC R&D offices using the Integrated Research Application System (IRAS). Patients will be enrolled once all required agreements are in place. Approximate figures for 2019 / 2020 indicate that 4,300 patients were admitted to hospitals with a heart attack in NI, with 40% of these patients having a STEMI (*n* = 1,720). 75% of these patients who had STEMI (*n* = 1,290) were treated in the Belfast region within the CCUs that will serve as the study centres (29,30). Thus, approximately 107 patients with STEMI will be available for recruitment across both study centres per month. Given the small sample size required, we have judged a 6-month period for recruitment to be sufficient (recruiting at least 7% of potential participants per month).

##### **Recruitment procedure**

A member of the clinical team (Nurse or Doctor) at each CCU will review the records of admitted patients with a screening questionnaire (see Appendix 3) to identify patients who satisfy the eligibility criteria for the study. Eligible patients will be approached by a member of the clinical team who will provide an information pack including an invitation to participate and patient information sheets (see Appendix 4). The member of the clinical team will verbally discuss the information sheets with the eligible patient and answer any questions before asking if the patient is willing to participate. The typical ‘cooling-off’ period (*i.e.,* one week) before making a decision regarding participation is not possible in this study as recruitment, intervention delivery, and data collection must occur before patient discharge from the CCU, which will ensure patients receive the intervention as soon as possible following the STEMI, and limit participant burden (*i.e.,* travelling to a recruitment appointment). A member of the clinical team will contact the Research Fellow to inform him of an interested potential participant. The Research Fellow will attend the ward to further discuss the study (*i.e.,* explaining the nature of an RCT and the potential for allocation to the control group) and answer any questions. If the patient is willing to participate, the Research Fellow will obtain informed consent (see Appendix 5) before randomising the participant and conducting baseline data collection. To set the results in context, informed consent will also be requested from participants for the extraction of information related to clinical characteristics (cardiac intervention received, prescribed medication, and co-morbidities) from their medical records. This study will seek adoption by the Northern Ireland Clinical Research Network (NICRN), which will involve research nurses extracting the required information from medical records of consenting participants. The research team will not have access to the medical records of patients. A private space will be used for recruitment and baseline data collection, such as an empty, private room at the CCU or a quiet area with a privacy screen. These procedures should take approximately 30 minutes.

###### ***i. Access and Generalisability***

This study has broad eligibility criteria and a simple recruitment strategy to allow ease of access for participants. A study log will be used by members of the clinical team and the Research Fellow to identify any bias or complications with screening and recruitment (see Appendix 6). Participant identification training will be delivered by the Research Fellow to the clinical team, with updates provided on any changes to the study protocol and eligibility criteria. A poster diagram of the recruitment procedure (see Appendix 7) will be displayed on each ward for reference by members of the clinical team. A £20 voucher will be provided to participants upon completion of the study as compensation for their time and any inconvenience caused by participation.

###### ***ii. Study Advertising and Accessibility of Participant Recruitment Information***

The PPI Group will advise on the development of all study documentation to ensure accessibility and adherence to plain English guidelines. We will accommodate potential participants with visual impairments by providing audio versions of documents as required. Study material will be translated from English if necessary.

###### ***iii. Randomisation Procedure***

An online randomisation service (random permuted blocks) provided by Sealed Envelope Ltd (31) will be used by the Research Fellow (GT) to randomise (1:1) patients to the intervention or control group. Randomisation will be conducted during the recruitment appointment after informed consent is obtained.

#### **Intervention Delivery**

##### ***Intervention Group***

CABIN (see Appendix 1) will be delivered in a single session to each participant (one-to-one) of the intervention group by the Research Assistant (MM) who is a cardiac nurse with over 20 years of clinical experience in CR. Following recruitment and randomisation, the Research Fellow will contact the Research Assistant to inform her of a participant who requires the intervention, his / her CCU location (Royal Victoria Hospital or Ulster Hospital), and planned discharge date. Subsequently, the Research Assistant will contact the clinical team at the relevant CCU to arrange a time to deliver the intervention to the participant when he / she is clinically stable, prior to discharge. The Research Assistant will use a staff-facing CABIN document (see Appendix 8) to guide intervention delivery, which will provide prompts (*i.e.,* relevant questions and actions) and space for notes. A private space will be used for intervention delivery, such as an empty, private room at the CCU or a quiet area with a privacy screen. Intervention delivery should take approximately 20 minutes.

##### ***Control Group***

As previously discussed, participants of the control group will receive a refined version of CABIN (see Appendix 2), which excludes psychological and emotional support discussions with a nurse and details some standard discharge information (information about CAD and stenting / stent placement). The Research Fellow will deliver (one-to-one) this refined version of CABIN prior to patient discharge from the CCU. He has a PhD in the area of CAD and CR, which provides him with the knowledge required to discuss the relevant information with control group participants. A private space will be used for intervention delivery, such as an empty, private room at the CCU or a quiet area with a privacy screen. Intervention delivery should take approximately 10 minutes.

#### **Outcome Measures**

##### **Feasibility Measurements and Process Evaluation (Objective 1)**

The primary objective of this study is to test the feasibility of the research design and CABIN delivery, which will inform the suitability of methods and procedures for a future effectiveness trial on a larger scale. Thus, the feasibility assessments and process evaluation discussed in the following sections represent processes that are key to the success of a larger study (32,33).

###### ***i. Reach (Recruitment Rate)***

The measurement of recruitment rate will allow the efficiency of the recruitment strategy to be assessed, with this information enabling the identification of potential issues and informing the required duration of the recruitment period for a larger study (34). The Research Fellow will maintain a record (see Appendix 9) of the number of patients screened for eligibility; the number eligible and invited to participate; and the number of patients who consented to participate per centre. When possible, the reasons for not entering the study will be recorded. Recruitment rate will be reported as the percentage of eligible patients who agreed to participate in the study, and the number of patients recruited monthly per centre over the recruitment period.

###### ***ii. Dose***

Dose will assess the completeness of intervention delivery, which will inform the feasibility of CABIN implementation. This information will be collected via an intervention checklist (see Appendix 10) that is completed by the Research Assistant following each intervention session.

###### ***iii. Fidelity***

Fidelity will be represented by adherence to the study protocol and procedures, which will identify any modifications required or issues to be resolved. This information will be collected via a study log (see Appendix 6) completed by the Research Fellow, Research Assistant, and members of the clinical team.

###### ***iv. Data Completeness***

The completeness of baseline and outcome measures will be determined as missing data may jeopardise the power of a future study (33). The Research Fellow will record details about data collection in the study log (see Appendix 6), which will highlight any problems or required changes to improve data collection for a future study (33). Data completeness will be reported as the percentage of recruited participants providing data for each baseline and outcome measure, with reasons for any missing data stated.

###### ***v. Acceptability, Context, and Possible Mechanisms of Impact***

A process evaluation of the research design / CABIN implementation will be conducted through measures of 1) Acceptability of intervention (*i.e.,* issues for development, required corrections, additional areas for inclusion, aspects enjoyed by participants, and barriers and facilitators to participation); 2) Context (*i.e.,* factors influencing study / intervention delivery and functioning, for instance, time and resources); and 3) Possible mechanisms of impact (*i.e.,* exploring how intervention activities may trigger change for participants). These measurements will be primarily assessed with qualitative data collected from exit interviews with participants and focus groups with CCU and CR staff. Information from study documents (*i.e.,* intervention checklist (Appendix 10), study log (Appendix 6), and post-study evaluation questionnaire (Appendix 11)) will supplement this qualitative data.

**Protocol for Exit Interviews with Participants**

**Design**

All participants (intervention and control group) will be eligible to complete a semi-structured interview (approximately 30 – 60 minutes in duration) at the final Time Point (TP) for data collection. A core set of questions in a “laddered style approach” will be used, which will ensure emerging ideas are explored (35). Interview questions for the intervention group participants (see Appendix 12) will focus on acceptability, context, and possible mechanisms of impact for intervention implementation and study delivery. As control group participants will not receive the full version of CABIN, their interview questions (see Appendix 13) will focus on acceptability of study delivery (*i.e.,* barriers and facilitators to participation / any recommendations or concerns).

**Procedure**

As part of the original consent process (see Appendix 5), patients will either agree or refuse to participate in the exit interview. For those who agree, the Research Fellow will conduct each interview individually with participants at the final TP for data collection, which will be held virtually (*i.e.,* Microsoft Teams) or face-to-face (*i.e.,* the participant’s home or a quiet meeting room at QUB) at the participant’s preference. All interviews will be audio-recorded before being transcribed verbatim by an external service. Data analysis of the transcripts will be performed iteratively (after each interview).

**Protocol for Focus Groups with Staff**

**Design**

CCU and CR staff will be invited to participate in virtual (*i.e.,* Microsoft Teams) focus groups (approximately 60 minutes in duration) at the end of the study. A core set of questions in a “laddered style approach” will be used, which will ensure emerging ideas are explored (35). These questions (see Appendix 14) will focus on acceptability, context, and possible mechanisms of impact for intervention implementation and study delivery.

**Participants**

CCU and CR staff at the collaborating hospital centres (Royal Victoria Hospital and Ulster Hospital) will be recruited.

**Inclusion Criteria**

1) Aged 18 and over.

2) Willing to provide informed consent.

**Exclusion Criteria**

1) Under the age of 18.

**Sample Size**

Four virtual focus groups across both centres (Royal Victoria Hospital, *n* = 2; Ulster Hospital, *n* = 2) will be conducted. Each focus group will consist of CCU (*n* = 3) and CR staff (*n* = 3). This sample size is based on recommendations in the literature for a study of this scope (38).

**Procedure**

The Research Fellow will conduct the focus groups at the end of the study. The Research Fellow will verbally discuss the focus groups with members of the research team who are clinical staff. These members will be asked to supply interested colleagues (CCU and CR staff) with an information pack including an invitation to participate and participant information sheets (see Appendix 15) for the focus groups. The participant information sheets will advise potential participants to consider their willingness to participate for one week (cooling-off period) before contacting the Research Fellow who will then answer any questions and inform the participant of the date for the virtual focus group. Informed consent (see Appendix 16) will be obtained electronically via Qualtrics from all participants before focus groups are conducted. Subsequently, participants will be asked to provide demographic details (age, gender, education level, marital status, occupation, and postcode (measure of socioeconomic background)) via Qualtrics (see Appendix 17), which will set the findings in context. All focus groups will be audio-recorded before being transcribed verbatim by an external service.

##### ***Preliminary Exploration of CABIN Impact (Objective 2)***

The secondary objective of this study is to perform a preliminary assessment of intervention effect on relevant outcomes, which will inform the selection of appropriate outcome measures for a larger, definitive study.

###### ***i. Questionnaires***

All participants will be asked to complete four short, validated questionnaires at each TP, which will provide information on the impact of CABIN on knowledge of CAD and CR; and psychological and emotional well-being:

1) CADE-Q SV (21): evaluates patients’ knowledge of CAD and core components of CR.

2) Brief Illness Perception Questionnaire (39): rapidly assesses the cognitive and emotional representations of illness.

3) The Hospital Anxiety and Depression Scale (40): detects states of depression and anxiety.

4) Personal Wellbeing Score (41): subjectively measures health status and health confidence.

These outcome measures were specifically chosen to minimise participant burden, whilst providing reliable data. As per advice from the PPI Group, the Research Fellow or Research Assistant will clearly explain the questionnaires to all participants before completion, and will be present to provide assistance, if required. The questionnaires will be compiled in a booklet (see Appendix 18), which will include a short cover sheet comprising questions concerning demographic details (age, gender, education level, employment status, marital status, and postcode (measure of socioeconomic background)). This demographic information will assist with setting the results in context. The cover sheet will be removed from the questionnaire booklet for the follow-up TPs. If a TP for data collection is held virtually, the participant will complete the questionnaire booklet via Qualtrics under the guidance of the Research Fellow. The completion of the questionnaire booklet will take approximately 20 minutes.

###### ***ii. CR Attendance***

The impact of CABIN on CR attendance will be investigated. Participants will be asked to provide informed consent (see Appendix 5) for the Research Fellow to be supplied with their CR attendance figures by the CR teams.

##### **Success Criteria**

A feasibility study should possess clearly defined *a priori* success criteria that are based on the outcome measures (32). These criteria serve as the basis for interpreting the results to ascertain the viability of conducting a larger, definitive study and / or to identify required modifications to promote success (33).

###### ***i. Reach (Recruitment Rate)***

Approximate figures for 2019 / 2020 indicate that 1,720 patients were admitted to hospitals with a STEMI in NI (29,30). Accordingly, for a future, multi-centre study including patients with STEMI across NI, a sample size of 315 would be required for a 5% margin of error, 95% confidence level, and a response distribution of 50%. Assuming a recruitment period of one year and five centres across the Health and Social Care Trusts in NI, the required monthly recruitment rate per centre would be approximately 6 participants. Therefore, for this study, a monthly recruitment rate of 6 participants per centre will be the success criterion.

###### ***ii. Data Completeness***

Missing data for > 20% of participants may pose a serious threat to validity (42). Therefore, data completeness will be deemed successful if data is received from ≥ 80% of participants at each TP (excluding qualitative data as sample sizes will be different).

###### ***iii. Acceptability of the Study Protocol and CABIN Implementation***

Acceptability of the study protocol and CABIN implementation (*i.e.,* issues for development, required corrections, aspects enjoyed by participants, and factors influencing study / intervention delivery) will be predominantly informed by the qualitative data collected form participants and staff. Success will constitute the provision of positive feedback on the study protocol and intervention delivery, along with no infeasible / major revisions being identified.

#### **Time Points for Data Collection**

Four TPs for data collection were chosen to provide detail of illness perception and emotional / psychological wellbeing for patients between discharge from the CCU and engagement with CR (see Table 1). All timing is from the point of STEMI diagnosis. TP-1 represents face-to-face recruitment and baseline data collection at the CCU before discharge, which will be conducted by the Research Fellow. TP-2 will constitute post-intervention data collection at the CCU before discharge, which will be performed by the Research Assistant (for intervention group) or Research Fellow (for control group) immediately following intervention delivery. Data collection at TP-3 and TP-4 coincides with the start and end of CR attendance, respectively. We will use these data points to assess engagement with CR across the duration of the programme. If a participant does not attend CR, the Research Fellow will calculate the same timescales and conduct data collection accordingly, with the same process occurring for those who reschedule their CR assessment. Data collection at TP-3 and TP-4 will be conducted virtually (*i.e.,* Microsoft Teams) or face-to-face (*i.e.,* the participant’s home or a private meeting room at QUB) depending on the participant’s preference. CCU and CR staff will be invited to participate in focus groups after data collection from patients has concluded.

##### ***Case Report Form***

The Research Fellow will record data collected at each TP on an pseudonymised Case Report Form (see Appendix 19), which will serve as a consolidated resource that outlines the data collected for each participant.

**Table 1.** Time Points for Data Collection.

| **Time Point** | **Time after diagnosis** | **Trigger** | **Location** | **Data collected** | **Duration (minutes)** | **Performed by** | |
| --- | --- | --- | --- | --- | --- | --- | --- |
| TP-1  (Baseline) | 12-96 hours | Pre-intervention (recruitment session) | CCU | 1) Clinical characteristics from medical records.  2) Questionnaire booklet (including demographic details). | 20 | NICRN Research Nurse (extracting clinical characteristics from medical records) and Research Fellow (questionnaire booklet) | |
| TP-2 | < 96 hours (before discharge from CCU) | Post-intervention | CCU | 1) Questionnaire booklet. | 20 | Research Assistant | Intervention Group |
|  |  |  |  |  |  | Research Fellow | Control Group |
| TP-3 | 3-4 weeks (may be different time frame depending on date of 1^st^ CR assessment) | 1^st^ CR assessment | Virtual or in-person | 1) Questionnaire booklet. | 20 | Research Fellow | |
| TP-4 | 13-14 weeks | Final CR assessment | Virtual or in-person | 1) Questionnaire booklet.  2) Post-study evaluation questionnaire.  3) Exit interview.  4) CR attendance. | 60 | Research Fellow | |
| Focus groups with staff | N/A | Completion of data collection from patients | Virtual | 1) Demographic details.  2) Qualitative data. | 60 | Research Fellow | |

TP = Time Point; CCU = Coronary Care Unit; NICRN = Northern Ireland Clinical Research Network; CR = Cardiac Rehabilitation; and N/A = Not Applicable.

### **Data Analysis**

#### **Quantitative Data Analysis**

Quantitative data will be organised and collated into one dataset in Microsoft Excel before being transferred to the Statistical Package for the Social Sciences (IBM SPSS Statistics, Version 28). Inferential statistics will not be performed as this study is not formally powered to detect statistical significance. The data will be reported with descriptive statistics. In terms of numerical data, continuous data will be presented as mean ± standard deviation, with discrete data reported as absolute numbers and percentages. Categorical data will be displayed as frequency / percentages. Within and between-group mean differences and 95% confidence intervals will be reported at each TP for the questionnaire data, with results interpreted according to minimum clinically important differences, if available.

#### **Qualitative Data Analysis**

The audio-recordings of semi-structured interviews and focus groups will be transcribed verbatim by an external service, with identifying information removed. Qualitative data will be analysed using the stages of the framework method of analysis; familiarisation, identifying a thematic framework, indexing, charting, mapping, and interpretation (43). The data in each group will be analysed separately to obtain an account for each experience. The research team will read and re-read transcripts for familiarity, with key issues and themes identified and a thematic framework developed. This framework will be applied to the transcripts and themes will be coded and charted, which will enable the refined themes to be mapped and interpreted to give a full account of the investigated phenomena. The accounts from each group will be compared to find similarities and differences between the groups. To enhance rigour, a process of member checking will be implemented, whereby the Research Fellow will give a descriptive summary and discuss the respective findings of each group with corresponding participants to ensure the analysis adequately reflects their accounts (44). Qualitative data analysis will be managed on NVivo (QSR International Pty Ltd. Release 1).

##### ***Integration of Quantitative and Qualitative data***

The quantitative and qualitative data will be integrated to achieve triangulation of findings (convergence of quantitative and qualitative data), which may generate a multidimensional understanding of the feasibility, theory, design, methods, implementation, and impact of CABIN (22). A triangulation protocol will be implemented according to the recommendations of Farmer et al. (45). The findings from the quantitative and qualitative data will be sorted and listed on the same page to ascertain if there is agreement, partial agreement, discrepancy / dissonance, or silence between them. Silence will represent a theme occurring in only one dataset. This assessment will be displayed in a convergence coding matrix, with the triangulated results being discussed by the research team for review and clarification. The reporting of this integrated data will comply with the ‘Good Reporting of a Mixed Methods Study’ framework (46).

## **Ethical Considerations**

This study will be conducted in accordance with Good Clinical Practice guidelines and adhere to the Declaration of Helsinki statement (1964). Ethical and governance approvals will be sought from QUB, NHS / HSC REC, and local HSC R&D offices (BHSCT and SEHSCT) using IRAS. The research team have prior experience with successful ethical applications and understand the regulations and timescales involved. This is a low-risk intervention delivered by an experienced team who are familiar with the processes required to obtain ethical and governance approvals, with no difficulties anticipated. We have consulted with Professor Mike Clarke (methodological expert), along with patients and staff in the development of this protocol to mitigate any areas of ethical concern. The intervention has been designed by patients and Cardiac Nurses who have provided their experience and expertise. We will liaise with NICHS if any significant changes to the protocol are required.

### ***Study Publicity***

To improve reach and exposure, we will advertise the study at ward-level. We will endeavour to maximise the profile of the study through publications and presentations, which will also raise awareness of NICHS and the support they provide.

### ***CCU Staff Training***

Medical and nursing staff will adhere to Trust Governance Requirements for Research Conduct and Procedure. The research team will provide training to all staff for identifying potential participants and the process for contacting the Research Fellow. We will equip clinical staff with a factsheet to answer commonly asked questions (see Appendix 20) and a visual representation of the recruitment process (see Appendix 7), which will assist with the implementation of the recruitment protocol. NICRN research nurses will receive a dedicated data collection form to facilitate extraction of clinical characteristics from medical records of consenting patients (see Appendix 21).

***Recruitment***

PPI input suggested that the research team need to spend time discussing the use of questionnaires to obtain measurements of emotional and psychological health. The Research Fellow will be explicit during the consent process to ensure the patient has fully understood the requirements of the study, which will equip him / her with the knowledge required to provide informed consent. This may include translating material for non-English speakers. The Research Fellow will explain the nature of an RCT and the potential for the participant to be randomised to the control group and receive only a limited version of the CABIN intervention. For those patients recruited to the control group, the Research Fellow will give them time to ask questions and refer any specific issues to the ward-based clinical team via the nurse in charge.

### ***CABIN***

Co-design methodology identified the need for more personalised information and psychological support in the acute phase following STEMI. The workshops were subsequently used to develop and refine CABIN based on patient experience, requirements, current scientific evidence, and guidance from the British Association for Cardiovascular Prevention and Rehabilitation (BACPR).

### ***Anonymity***

The identification of eligible patients will be completed by professional gatekeepers (Nurse or Doctor). These individuals will make primary contact with the patient about the study and determine their willingness to be involved. As such, the research team will not see any records or have any contact with patients before the Research Fellow meets with them to obtain informed consent. Medical records will only be assessed by the NICRN research nurses to obtain clinical characteristics if written informed consent is obtained from participants. Each participant will be assigned a Personal Identification Number (PIN), which will replace identifying information within datasets, such as: name and hospital Health & Care Numbers.

### ***Burden***

Study materials have been chosen / created to minimise participant burden. All patient information sheets and informed consent forms are user friendly in design and have been evaluated by the PPI Group. All study procedures will be conducted at a private space, for instance, in a quiet room at a CCU or QUB, the participant’s home, or virtually (*i.e.,* Microsoft Teams). The questionnaires selected take a short time to complete and are validated, which aims to reduce participant burden. The location of data collection in the later stages of the study will be determined by participant preference. We will provide a £20 voucher to each participant upon study completion as compensation for data usage, time commitment, and any inconvenience caused.

### ***Distress***

Participant distress may occur during the study (*i.e.,* when completing questionnaires or discussing sensitive topics in an interview). As such, a distress protocol (see Appendix 22) has been developed by the research team, which will be implemented in the event of a participant becoming upset. Moreover, to support distressed participants, the research team will request for NICHS to provide additional information if required, for instance, lifestyle leaflets. This technique was used in previous research, which the participants found helpful.

### ***Safeguarding***

Patient participants who demonstrate low overall scores on the included questionnaires and scales will be immediately highlighted by the Research Fellow to the healthcare team. This will constitute an effort to identify those patients in need of additional support or follow-up. It will then be at the discretion of the healthcare team as to what extra support is provided to these individuals. If an interview occurs at a participant’s home, the Research Fellow will comply with the Lone Working policy at QUB for his safety.

### ***Study Withdrawal***

Participants will be informed of the voluntary nature of their inclusion in this research and their ability to withdraw at any point, without compromising their current clinical care. As stated in the patient information sheets, if a participant withdraws 2 weeks after the time of initial data collection, his / her data may be used for analysis (with the participant’s consent). The reason for withdrawal will be noted for review.

### ***Data Protection and Management***

The requirements of the General Data Protection Regulation and Data Protection Act (2018) will be adhered to, as per the QUB Data Protection Policy. All information will be stored in a secure manner in compliance with local Trust governance requirements. We will only acquire information that is required to undertake the study. All paper data will be stored in a Master File and Site Files in locked filing cabinets, inside locked offices within the School of Nursing and Midwifery, QUB and the collaborating centres (BHSCT and SEHSCT), respectively. All electronic data will be stored on an encrypted and password protected computer, inside a locked office within the School of Nursing and Midwifery, QUB. Individual data files will also be password protected. All audio-recordings will be destroyed after the transcripts have been checked for accuracy.

Personal data will be pseudonymised. A PIN system will be used, whereby each participant is allocated a unique number. This number will replace identifying information within datasets, such as: name and Health & Care Number (used within the hospital). This will help preserve anonymity when the data is reviewed by the research team. A master list that links PINs to patients’ personal information (*i.e.,* name and contact details) will be stored securely and separately from other data in a locked office within the School of Nursing and Midwifery, QUB. The researcher will endeavour to use a participant’s first name only during audio-recordings of interviews and focus groups. Transcriptions of audio-recordings will be redacted and transferred through secure processes (*i.e.,* linked with PIN). We will only use trusted staff who have completed a Data Sharing Agreement with the University for transcribing audio-recordings. All data presented in publications will be anonymised to protect participant confidentiality.

Consent will be obtained from participants for data preservation and sharing in an anonymised format. As per the QUB Research Data Management Policy, data will be retained in an anonymised format for a minimum of 5 years following publication if they are of continuing value to the researcher and the wider research community. Upon project completion, data sets will be allocated a DOI and transferred to the University’s research information management system (Pure) for long-term preservation, which will contain links to the project and relevant publications. All data that may identify participants will be destroyed following University procedures. Data will be exclusive to the research team until the completion of the study and publication of findings. Data generated by the project may be shared in an anonymised format after completion. The Principal Investigator (PI, Professor Donna Fitzsimons) will make the decision about whether to supply research data to a potential new user. Before data is shared, a data-sharing agreement will be issued and signed by appropriate authorities, which will prohibit any attempt by external users to (a) identify study participants form the released data or otherwise breach confidentiality and (b) make unapproved contact with any study participants. The MRC Policy and Guidance on Sharing of Research Data from Population and Patient Studies will be adhered to. The PI will be the first point of contact for all queries in relation to the data and will also have overall responsibility for the production and maintenance of metadata. The Research Fellow will have responsibility for study-wide data management, metadata creation, data security, and quality assurance of data. Preparation and upload of the data will be carried out by the research team with the support of QUB Information Services staff.

## **Dissemination**

The protocol / methods and results of this study will be disseminated via publications in peer-reviewed journals and scientific conferences (European Society of Cardiology - Preventative Cardiology Congress, EuroHeartCare, Irish Cardiac Society, and BACPR). We will link with NICHS to publicise the study and increase awareness of both NICHS and the research it supports. We will also produce posters possessing lay terminology in relation to the study purpose and methodology (during data collection phase) and similar posters displaying the results, impact, and future plans. These posters will be suitable for online viewing whilst ensuring accessibility for those with disabilities. We will utilise opportunities within QUB to disseminate the study methods and results, for instance, post-doc events and the School of Nursing Annual Conference. Members of the PPI Group (*i.e.,* patients) will be involved with dissemination of results and co-authorship of papers/abstracts where possible.

## **Potential Impact**

If feasible and acceptable, this research will progress to ‘Evaluation’, which is the next phase of the MRC framework for developing complex interventions (20). This will involve performing a definitive, multi-centre RCT to assess the effectiveness of CABIN in a real-world setting. Ultimately, this research could lead to the implementation of CABIN in routine clinical practice,

which may increase uptake to CR and improve outcomes for cardiac patients, such as: a reduction in recurrent cardiac events and lower readmission rates for anxiety-related chest pain. This intervention may also be replicated for other cardiac conditions and beyond the specialty into respiratory or stroke patients who face similar psychological challenges post-diagnosis. Importantly, the high degree of personalisation within CABIN may enhance the health literacy of patients and empower self-management of their conditions in partnership with local support network and agencies (*i.e.,* NICHS).

## **Management Plan**

Abbreviations: DF = Donna Fitzsimons, JB= Judy Bradley, PD = Patrick Donnelly, GC = Gemma Caughers, GT = Gareth Thompson, MM = Maria Mooney, MC = Mike Clarke, and CMcK = Clare McKeaveney.

- DF is the PI and is an experienced cardiovascular nurse and researcher who will provide oversight and be primarily responsible for study delivery (alternative PI to deputise if and when required).
- JB is the alternative PI and has co-authored the proposal and is an intrinsic member of the team. JB can assume the responsibility of PI if required with no negative impact to the study. Her role is also to provide additional oversight and link with clinical teams in the BHSCT.
- PD has contributed to the proposal and as a clinical cardiologist will advise on the delivery of the study. PD will assist in identifying potential participants and support with raising the study profile within the Cardiology clinical teams at the SEHSCT.
- GT is the Research Fellow who will conduct the daily running of the study, such as: training staff for potential participant identification, recruiting patients, data collection, administrative tasks, analysis, write-up, and dissemination of findings.
- GC led the completion of the protocol and is an experienced cardiovascular nurse and researcher. GC can assume the responsibility of the Research Fellow or Research Assistant if required with no negative impact to the study.
- MM is the Research Assistant and is a cardiac nurse with over 20 years of clinical experience. MM will be responsible for the safe delivery of the intervention and conducting post-intervention measurements on the intervention group.
- MC and CMcK are methodological and statistical experts who will provide guidance and advice over the course of the study.
- The team will meet regularly for updates and quarterly reports will be sent to NICHS. NICHS will also be updated at key points within the study, for instance, starting recruitment and achieving desired sample size. The Research Fellow will immediately report any safety issues or concerns to the team and escalate where appropriate.

### ***Contingency Plan***

All members of the team have full understanding of the study and associated requirements. GC will be trained in all aspects of study / intervention delivery should the Research Fellow or Research Assistant be unavailable in emergency circumstances. The analysis and writing of results can be transferred within the team, if necessary. We will inform NICHS if there are any potential or actual difficulties in delivering the project.

## **Project Milestones**

**Table 2.** Project Milestones

| **Date** | **Milestone** | **Project Month** | **Person Responsible** |
| --- | --- | --- | --- |
| November 2022 - March 2023 | Ethics and Governance Approval. | 0 | Research Fellow |
| April 2023 | Publicising Study Initiation. | 1 | Entire Team |
| April 2023 and May 2023 | CCU Staff Training. | 1 & 2 | Research Fellow |
| April 2023 – June 2023 | Writing Methodology Paper. | 1 - 3 | Entire Team |
| May 2023 | Recruitment Start. | 2 | Research Fellow |
| May 2023 – October 2023 | Recruitment Open. | 2 - 7 | Research Fellow |
| May 2023 – October 2023 | Baseline Data Collection (TP-1) and Intervention Delivery. | 2 - 7 | Research Fellow (data collection) and Research Assistant (intervention delivery) |
| May 2023 – October 2023 | Post-Intervention Data Collection (TP-2). | 2-7 | Research Assistant |
| June 2023 – November 2023 | TP-3 Data Collection (Start of CR). | 3 - 8 | Research Fellow |
| July 2023 – January 2024 | TP-4 Data Collection (End of CR). | 4 - 9 | Research Fellow |
| January 2024 – February 2024 | Focus Groups with Staff. | 9 & 10 | Research Fellow |
| July 2023 – August 2023 | Conference Abstract Writing. | 4 & 5 | Entire Team |
| February 2024 – April 2024 | Refinement of CABIN for Further Testing. | 10 - 12 | Entire Team |
| February 2024 – April 2024 | Results Papers. | 10 - 12 | Entire Team |
| April 2024 | Conference Presentations. | 12 | Entire Team |

CCU = Coronary Care Unit, TP = Time Point, CR = Cardiac Rehabilitation, and CABIN = Cardiac brief intervention.

## **Project Deliverables**

**Table 3.** Project Deliverables.

| **Deliverable** | **Context** | **Month of Delivery** | **Person Responsible** |
| --- | --- | --- | --- |
| Methodology / Protocol Paper | Publication in peer-reviewed scientific journal. | 3 | Research Fellow and review by team. |
| Abstract Submission (2 European Conferences) | Abstract for conference poster / presentation. | 6 | Research Fellow and review by team. |
| Abstract Submission (1 local conference) | Abstract for conference poster / presentation. | 8 | Research Fellow and review by team. |
| Results Paper | Publication in peer-reviewed scientific journal. | 12 | Research Fellow and review by team. |
| Abstract Submission (BACPR) | Abstract for conference poster / presentation. | 12 | Research Fellow and review by team. |
| Dissemination of results via NICHS, social media, HSC trust research departments, and print media. | Dissemination of findings. | 11 - 12 | Research Fellow. |

BACPR = British Association for Cardiovascular Prevention and Rehabilitation, NICHS = Northern Ireland Chest, Heart, and Stroke, and HSC = Health and Social Care.

##
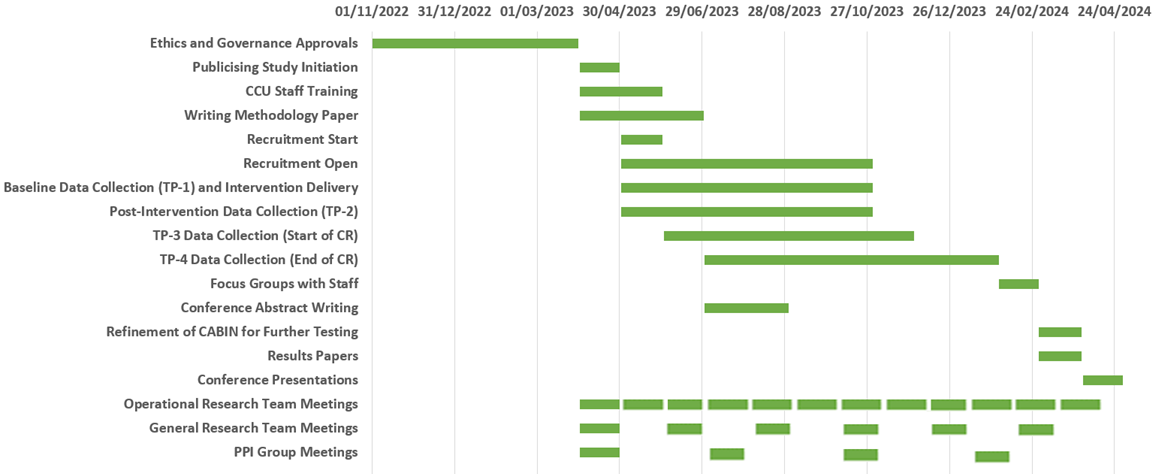
**Project Timeline**

## **Reference List**

1. Dullaghan L, Lusk L, McGeough M, Donnelly P, Herity N, Fitzsimons D. ‘I am still a bit unsure how much of a heart attack it really was!’ Patients presenting with non ST elevation myocardial infarction lack understanding about their illness and have less motivation for secondary prevention. *European Journal of Cardiovascular Nursing*. 2014;13(3):270–6. Available from: <https://academic.oup.com/eurjcn/article/13/3/270/5933197>

2. McAnirn G, Lusk L, Donnelly P, McKenna N, Hamill C, Fitzsimons D. Shocked and guilty, but highly motivated: Younger patients’ experience post MI. *British Journal of Cardiac Nursing*. 2015;10(12):610–5. Available from: <https://www.magonlinelibrary.com/doi/10.12968/bjca.2015.10.12.610>

3. Herber OR, Smith K, White M, Jones MC. ‘Just not for me’ – contributing factors to nonattendance/noncompletion at phase III cardiac rehabilitation in acute coronary syndrome patients: a qualitative enquiry. *J Clin Nurs*. 2017;26(21–22):3529–42. Available from: <https://onlinelibrary.wiley.com/doi/full/10.1111/jocn.13722>

4. Dalal HM, Doherty P, Taylor RS. Cardiac rehabilitation. *BMJ.* 2015;351. Available from: <https://www.bmj.com/content/351/bmj.h5000>

5. McGrady A, Burkes R, Badenhop D, McGinnis R. Effects of a Brief Intervention on Retention of Patients in a Cardiac Rehabilitation Program. *Applied Psychophysiology Biofeedback*. 2014;39(3–4):163–70. Available from: <https://link.springer.com/article/10.1007/s10484-014-9252-y>

6. Clark M, Kelly T, Deighan C. A Systematic Review of the Heart Manual Literature. European *Journal of Cardiovascular Nursing*. 2011;10(1):3–13. Available from: <https://academic.oup.com/eurjcn/article/10/1/3/5929316>

7. Wakefield B, Drwal K, Scherubel M, Klobucar T, Johnson S, Kaboli P. Feasibility and Effectiveness of Remote, Telephone-Based Delivery of Cardiac Rehabilitation. *Telemedicine and e-Health*. 2013;20(1):32–8. Available from: <https://www.liebertpub.com/doi/10.1089/tmj.2013.0079>

8. Neubeck L, Redfern JU, Fernandez R, Briffa T, Bauman A, Freedman S ben. Telehealth interventions for the secondary prevention of coronary heart disease: a systematic review. *European Journal of Cardiovascular Prevention and Rehabilitation*. 2009;16(3):281–9. Available from: <https://academic.oup.com/eurjpc/article/16/3/281/5931930>

9. Wongvibulsin S, Habeos EE, Huynh PP, Xun H, Shan R, Porosnicu Rodriguez KA, et al. Digital Health Interventions for Cardiac Rehabilitation: Systematic Literature Review. *J Med Internet Res*. 2021;23(2):e18773. Available from: <https://www.jmir.org/2021/2/e18773>

10. National Audit of Cardiac Rehabilitation. *NACR Quality and Outcomes Report 2019.* [https://www.bhf.org.uk/informationsupport/publications/statistics/national-audit-of-cardiac-rehabilitation-quality-and-outcomes-report-2019#](https://www.bhf.org.uk/informationsupport/publications/statistics/national-audit-of-cardiac-rehabilitation-quality-and-outcomes-report-2019). 2019.

11. Hendriks JML, Gallagher C, Astley C, Linz D, Gallagher R. Cardiac rehabilitation services: A global perspective on performance and barriers. *Int J Cardiol Heart Vasc*. 2019;24:100410. Available from: <https://doi.org/10.1016/j.ijcha.2019.100410>

12. Caughers G, Bradley J, Donnelly P, Fitzsimons D. Co-design of cardiac rehabilitation delivery in the aftermath of COVID-19, putting the patient view back in the picture. *European Journal of Cardiovascular Nursing*. 2021;20(Supplement_1). Available from: <https://academic.oup.com/eurjcn/article/20/Supplement_1/zvab060.068/6330374>

13. Chindhy S, Taub PR, Lavie CJ, Shen J. Current challenges in cardiac rehabilitation: strategies to overcome social factors and attendance barriers.
*Expert Review of Cardiovascular Therapy*. 2020 ;18(11):777–89. Available from: <https://www.tandfonline.com/doi/abs/10.1080/14779072.2020.1816464>

14. Daly J, Sindone AP, Thompson DR, Hancock K, Chang E, Davidson P. Barriers to participation in and adherence to cardiac rehabilitation programs: a critical literature review. *Prog Cardiovasc Nurs*. 2002;17(1):8–17. Available from: <https://pubmed.ncbi.nlm.nih.gov/11872976/>

15. Figueiras MJ, Maroco J, Monteiro R, Caeiro R, Dias Neto D. Randomized controlled trial of an intervention to change cardiac misconceptions in myocardial infarction patients.
*Psychology, Health & Medicine*. 2016;22(3):255–65. Available from: <https://www.tandfonline.com/doi/abs/10.1080/13548506.2016.1153677>

16. McPhillips R, Salmon P, Wells A, Fisher P. Cardiac Rehabilitation Patients’ Accounts of Their Emotional Distress and Psychological Needs: A Qualitative Study. *J Am Heart Assoc*. 2019;8(11). Available from: <https://www.ahajournals.org/doi/abs/10.1161/JAHA.118.011117>

17. Michie S, O’Connor D, Bath J, Giles M, Earll L. Cardiac rehabilitation: The psychological changes that predict health outcome and healthy behaviour.
*Psychology, Health & Medicine*. 2010;10(1):88–95. Available from: <https://www.tandfonline.com/doi/abs/10.1080/13548500512331315398>

18. Caughers G, Bradley J, Fitzsimons D, Donnelly P. *Rejuvenating Rehabilitation – Outcomes from an Experience Based Co-Design approach to improve engagement with Cardiac Rehabilitation.* Poster session presented at British Association of Cardiovascular Prevention and Rehabilitation, Belfast, United Kingdom. Available from: <https://pure.qub.ac.uk/en/publications/rejuvenating-rehabilitation-outcomes-from-an-experience-based-co->

19. Richards SH, Anderson L, Jenkinson CE, Whalley B, Rees K, Davies P, et al. Psychological interventions for coronary heart disease: Cochrane systematic review and meta-analysis. *Eur J Prev Cardio*. 2018;25(3):247–59. Available from: <https://academic.oup.com/eurjpc/article/25/3/247/5926174>

20. Skivington K, Matthews L, Simpson SA, Craig P, Baird J, Blazeby JM, et al. Framework for the development and evaluation of complex interventions: gap analysis, workshop and consultation-informed update. *Health Technol Assess*. 2021;25(57):1–132. Available from: <https://doi.org/10.3310/hta25570>

21. Ghisi GL de M, Sandison N, Oh P. Development, pilot testing and psychometric validation of a short version of the coronary artery disease education questionnaire: The CADE-Q SV. *Patient Educ Couns.* 2016;99(3):443–7. Available from: <https://doi.org/10.1016/j.pec.2015.11.002>

22. Dickson VV, Page SD. Using mixed methods in cardiovascular nursing research: Answering the why, the how, and the what’s next. *European Journal of Cardiovascular Nursing*. 2021;20(1):82–9. Available from: <https://academic.oup.com/eurjcn/article/20/1/82/6132767>

23. Cameron LD, Leventhal H. *The self-regulation of health and illness behaviour*. London: Routledge; 2003.

24. Eldridge SM, Chan CL, Campbell MJ, Bond CM, Hopewell S, Thabane L, et al. CONSORT 2010 statement: extension to randomised pilot and feasibility trials. *BMJ*. 2016;355. Available from: <https://www.bmj.com/content/355/bmj.i5239>

25. National Institute for Health and Care Research. *Being Inclusive in Public Involvement in Health and Care Research*. <https://www.nihr.ac.uk/documents/being-inclusive-in-public-involvement-in-health-and-care-research/27365#What_is_public_involvement>?. 2021.

26. Billingham SA, Whitehead AL, Julious SA. An audit of sample sizes for pilot and feasibility trials being undertaken in the United Kingdom registered in the United Kingdom Clinical Research Network database. *BMC Med Res Methodol*. 2013;13(1):1–6. Available from: <https://link.springer.com/articles/10.1186/1471-2288-13-104>

27. Murphy PJ, Sharry JM, Casey D, Doherty S, Gillespie P, Jaarsma T, et al. Sexual counselling for patients with cardiovascular disease: protocol for a pilot study of the CHARMS sexual counselling intervention. *BMJ Open*. 2016;6(6):e011219. Available from: <https://bmjopen.bmj.com/content/6/6/e011219>

28. Higgins RO, Rogerson M, Murphy BM, Navaratnam H, Butler M v., Barker L, et al. Cardiac Rehabilitation Online Pilot: Extending Reach of Cardiac Rehabilitation. *Journal of Cardiovascular Nursing*. 2017;32(1):7–13. Available from: <https://journals.lww.com/jcnjournal/Fulltext/2017/01000/Cardiac_Rehabilitation_Online_Pilot__Extending.3.aspx>

29. British Heart Foundation. *Heart and Circulatory Disease Statistics: Chapter 2 – Morbidity.* <https://www.bhf.org.uk/what-we-do/our-research/heart-statistics/heart-statistics-publications/cardiovascular-disease-statistics-2021>. 2021.

30. Myocardial Ischaemia National Audit Project. *2019 Summary Report.* <https://www.hqip.org.uk/resource/myocardial-ischaemia-national-audit-project-minap-2019-summary-report/#.Y0_rYPzMK3B>. 2019.

31. Sealed Envelope Ltd. *Simple Randomisation Service.* <https://www.sealedenvelope.com/simple-randomiser/v1/>. 2022.

32. Thabane L, Ma J, Chu R, Cheng J, Ismaila A, Rios LP, et al. A tutorial on pilot studies: The what, why and how*. BMC Med Res Methodol*. 2010;10(1):1–10. Available from: <https://bmcmedresmethodol.biomedcentral.com/articles/10.1186/1471-2288-10-1>

33. Avery KNL, Williamson PR, Gamble C, Francischetto EOC, Metcalfe C, Davidson P, et al. Informing efficient randomised controlled trials: exploration of challenges in developing progression criteria for internal pilot studies. *BMJ Open*. 2017;7(2):e013537. Available from: <https://bmjopen.bmj.com/content/7/2/e013537>

34. Moore CG, Carter RE, Nietert PJ, Stewart PW. Recommendations for Planning Pilot Studies in Clinical and Translational Research. *Clin Transl Sci*. 2011;4(5):332–7. Available from: <https://onlinelibrary.wiley.com/doi/full/10.1111/j.1752-8062.2011.00347.x>

35. Price B. Laddered questions and qualitative data research interviews. *J Adv Nurs*. 2002;37(3):273–81. Available from: <https://onlinelibrary.wiley.com/doi/full/10.1046/j.1365-2648.2002.02086.x>

36. Braun V, Clarke V. Using thematic analysis in psychology. *Qual Res Psychol*. 2006;3(2):77–101. DOI: [10.1191/1478088706qp063oa](https://doi.org/10.1191/1478088706qp063oa)

37. Braun V, Clarke V. *Successful qualitative research: A practical guide for beginners*. London (UK): SAGE Publications Limited; 2013.

38. Hennink M, Kaiser BN. Sample sizes for saturation in qualitative research: A systematic review of empirical tests. *Soc Sci Med*. 2022;292:114523. Available from: <https://doi.org/10.1016/j.socscimed.2021.114523>

39. Broadbent E, Petrie KJ, Main J, Weinman J. The Brief Illness Perception Questionnaire. *J Psychosom Res*. 2006;60(6):631–7. Available from: <https://doi.org/10.1016/j.jpsychores.2005.10.020>

40. Zigmond AS, Snaith RP. The Hospital Anxiety and Depression Scale. *Acta Psychiatr Scand*. 1983;67(6):361–70. Available from: <https://onlinelibrary.wiley.com/doi/full/10.1111/j.1600-0447.1983.tb09716.x>

41. Benson T, Sladen J, Liles A, Potts HWW. Personal Wellbeing Score (PWS)—a short version of ONS4: development and validation in social prescribing. *BMJ Open Qual*. 2019;8(2):e000394. Available from: <https://bmjopenquality.bmj.com/content/8/2/e000394>

42. Higgins JPT, Savović J, Page MJ, Elbers RG, Sterne JAC. Assessing risk of bias in a randomized trial. *Cochrane Handbook for Systematic Reviews of Interventions*. 2019;205–28. Available from: <https://onlinelibrary.wiley.com/doi/full/10.1002/9781119536604.ch8>

43. Pope C. Qualitative research in health care: Analysing qualitative data. *BMJ*. 2000;320(7227):114–6. Available from: <https://doi.org/10.1136/bmj.320.7227.114>

44. Polit D, Beck C. *Nursing research: Generating and assessing evidence for nursing practice.* 9th ed. London (UK): Lippincott Williams & Wilkins; 2012.

45. Farmer T, Robinson K, Elliott SJ, Eyles J. Developing and Implementing a Triangulation Protocol for Qualitative Health Research. *Qual Health Res*. 2006;16(3):377–94. Available from: [https://doi.org/10.1177/1049732305285](https://doi.org/10.1177/1049732305285708)

46. O’Cathain A, Murphy E, Nicholl J. The Quality of Mixed Methods Studies in Health Services Research. *J Health Serv Res Policy*. 2008;13(2):92–8. Available from: <https://doi.org/10.1258/jhsrp.2007.007074>
