# Supplementary material for: The feasibility of delivering cardiac brief intervention to patients following ST-elevation myocardial infarction: Protocol for a pilot randomised controlled trial
Source: PLoS One. 2024 Jul 2;19(7):e0306406. doi: 10.1371/journal.pone.0306406 (PMC11218979; doi:10.1371/journal.pone.0306406)
Supplement: S4 Text — (PDF) [file pone.0306406.s007.pdf]

Health and Social Care Research Ethics Committee A (HSC REC A)
Email: reca@hscni.net

03 April 2023

Professor Donna Fitzsimons
Head of School of Nursing and Midwifery
Queen's University of Belfast
School of Nursing and Midwifery,
Medical Biology Centre,
97 Lisburn Road, Belfast, Northern Ireland.
BT9 7BL

Dear Professor Fitzsimons

**Study title:**	**CABIN – (CArdiac Brief INtervention): a feasibility study to promote engagement with cardiac rehabilitation through an early, personalised, and holistic intervention.**
**REC reference:**	**23/NI/0032**
**Protocol number:**	**N/A**
**IRAS project ID:**	**321747**

Thank you for your letter of , responding to the Research Ethics Committee's (REC) request for further information on the above research and submitting revised documentation.

The further information has been considered on behalf of the Committee by the Chair.

Confirmation of ethical opinion

On behalf of the Committee, I am pleased to confirm a favourable ethical opinion for the above research on the basis described in the application form, protocol and supporting documentation as revised, subject to the conditions specified below.

Good practice principles and responsibilities

The UK Policy Framework for Health and Social Care Research sets out principles of good practice in the management and conduct of health and social care research. It also outlines the responsibilities of individuals and organisations, including those related to the four elements of research transparency:

1. registering research studies
2. reporting results
3. informing participants
4. sharing study data and tissue

Conditions of the favourable opinion

The REC favourable opinion is subject to the following conditions being met prior to the start of the study.

Confirmation of Capacity and Capability (in England, Northern Ireland and Wales) or NHS management permission (in Scotland) should be sought from all NHS organisations involved in the study in accordance with NHS research governance arrangements. Each NHS organisation must confirm through the signing of agreements and/or other documents that it has given permission for the research to proceed (except where explicitly specified otherwise).

Guidance on applying for HRA and HCRW Approval (England and Wales)/ NHS permission for research is available in the Integrated Research Application System.

For non-NHS sites, site management permission should be obtained in accordance with the procedures of the relevant host organisation.

Sponsors are not required to notify the Committee of management permissions from host organisations

Registration of Clinical Trials

All research should be registered in a publicly accessible database and we expect all researchers, research sponsors and others to meet this fundamental best practice standard.

It is a condition of the REC favourable opinion that **all clinical trials are registered** on a publicly accessible database within six weeks of recruiting the first research participant. For this purpose, 'clinical trials' are defined as:

- clinical trial of an investigational medicinal product
- clinical investigation or other study of a medical device
- combined trial of an investigational medicinal product and an investigational medical device
- other clinical trial to study a novel intervention or randomised clinical trial to compare interventions in clinical practice.

Failure to register a clinical trial is a breach of these approval conditions, unless a deferral has been agreed by the HRA (for more information on registration and requesting a deferral see: Research registration and research project identifiers).

If you have not already included registration details in your IRAS application form you should notify the REC of the registration details as soon as possible.

Publication of Your Research Summary

We will publish your research summary for the above study on the research summaries section of our website, together with your contact details, no earlier than three months from the date of this favourable opinion letter.

Should you wish to provide a substitute contact point, make a request to defer, or require further information, please visit: https://www.hra.nhs.uk/planning-and-improving-research/application-summaries/research-summaries/

N.B. If your study is related to COVID-19 we will aim to publish your research summary within 3 days rather than three months.

During this public health emergency, it is vital that everyone can promptly identify all relevant research related to COVID-19 that is taking place globally. If you haven't already done so, please register your study on a public registry as soon as possible and provide the REC with the registration detail, which will be posted alongside other information relating to your project. We are also asking sponsors not to request deferral of publication of research summary for any projects relating to COVID-19. In addition, to facilitate finding and extracting studies related to COVID-19 from public databases, please enter the WHO official acronym for the coronavirus disease (COVID-19) in the full title of your study. Approved COVID-19 studies can be found at: https://www.hra.nhs.uk/covid-19-research/approved-covid-19-research/

It is the responsibility of the sponsor to ensure that all the conditions are complied with before the start of the study or its initiation at a particular site (as applicable).

After ethical review: Reporting requirements

The attached document "After ethical review – guidance for researchers" gives detailed guidance on reporting requirements for studies with a favourable opinion, including:

- Notifying substantial amendments
- Adding new sites and investigators
- Notification of serious breaches of the protocol
- Progress and safety reports
- Notifying the end of the study, including early termination of the study
- Final report
- Reporting results

The latest guidance on these topics can be found at https://www.hra.nhs.uk/approvals-amendments/managing-your-approval/.

Ethical review of research sites

Non-NHS/HSC sites

I am pleased to confirm that the favourable opinion applies to any non-NHS/HSC sites listed in the application, subject to site management permission being obtained prior to the start of the study at the site.

Approved documents

The final list of documents reviewed and approved by the Committee is as follows:

Document	*Version*	*Date*
Copies of materials calling attention of potential participants to the research [Ward Advertisement Poster]	1	11 November 2022
Covering letter on headed paper [Covering letter]	1	01 December 2022
Evidence of Sponsor insurance or indemnity (non NHS Sponsors only) [Indemnity Certificate_QUB]	1	13 February 2023
Interview schedules or topic guides for participants [Exit interviews with intervention group participants]	1	11 November 2022
Interview schedules or topic guides for participants [Exit interviews with control group participants]	1	11 November 2022

Interview schedules or topic guides for participants [Guide for focus groups with staff]	1	11 November 2022
IRAS Application Form [IRAS_Form_14022023]		14 February 2023
IRAS Checklist XML [Checklist_31032023]		31 March 2023
Letter from funder		
Letter from sponsor [Sponsorship letter_QUB]	1	13 February 2023
Letters of invitation to participant [Invitation to Participate - Patients]	1	11 November 2022
Letters of invitation to participant [Invitation to Participate - Staff]	1	11 November 2022
Non-validated questionnaire [Screening questionnaire]	1	11 November 2022
Non-validated questionnaire [Post-study evaluation questionnaire]	1	11 November 2022
Non-validated questionnaire [Demographic questionnaire for staff]	1	11 November 2022
Other [Study Log]	1	11 November 2022
Other [Poster diagram of recruitment process for clinical staff]	1	11 November 2022
Other [Staff-facing CABIN document]	1	11 November 2022
Other [Recruitment log]	1	11 November 2022
Other [Intervention checklist]	1	11 November 2022
Other [Case Report Form]	1	11 November 2022
Other [Factsheet for clinical staff]	2	31 January 2023
Other [Clinical Characteristics Data Extraction Form]	1	31 January 2023
Other [Distress protocol]	1	11 November 2022
Other [CABIN Leaflet_Control Group]	1	16 December 2022
Other [CABIN Leaflet _Intervention Group]	1	16 December 2022
Other [Response Letter]	1	31 March 2023
Participant consent form [Consent Form - Patient]	3	31 March 2023
Participant consent form [Consent Form - Staff]	3	31 March 2023
Participant information sheet (PIS) [Participant Information Sheets - Patients]	3	31 March 2023
Participant information sheet (PIS) [Participant Information Sheets - Staff]	3	31 March 2023
Research protocol or project proposal [Study Protocol]	2	20 January 2023
Summary CV for Chief Investigator (CI) [Summary CV for Chief Investigator]	1	11 November 2022
Summary, synopsis or diagram (flowchart) of protocol in non technical language [Study flow diagram]	2	30 January 2023
Validated questionnaire [Questionnaire booklet]	1	11 November 2022

Statement of compliance

The Committee is constituted in accordance with the Governance Arrangements for Research Ethics Committees and complies fully with the Standard Operating Procedures for Research Ethics Committees in the UK.

User Feedback

The Health Research Authority is continually striving to provide a high quality service to all applicants and sponsors. You are invited to give your view of the service you have received and the application procedure. If you wish to make your views known please use the feedback form available on the HRA website: http://www.hra.nhs.uk/about-the-hra/governance/quality-assurance/

HRA Learning

We are pleased to welcome researchers and research staff to our HRA Learning Events and online learning opportunities – see details at: https://www.hra.nhs.uk/planning-and-improving-research/learning/

**IRAS project ID: 321747**	**Please quote this number on all correspondence**

With the Committee's best wishes for the success of this project.

Yours sincerely

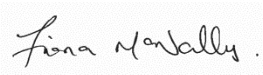

pp Dr Alastair Walker **Chair of meeting**

Email:RECA@hscni.net

Enclosures:	"After ethical review – guidance for researchers" *[SL-AR2]*
Copy to:	Ms Paula Tighe
	Northern Ireland: research.gateway@hscni.net
