# Supplementary material for: The feasibility of delivering cardiac brief intervention to patients following ST-elevation myocardial infarction: Protocol for a pilot randomised controlled trial
Source: PLoS One. 2024 Jul 2;19(7):e0306406. doi: 10.1371/journal.pone.0306406 (PMC11218979; doi:10.1371/journal.pone.0306406)
Supplement: S5 Text — (DOCX) [file pone.0306406.s008.docx]

29 May 2024

| NICHS project code | **2021_H07** |
| --- | --- |
| Grant Holder (PI) | Professor Donna Fitzsimons |
| named alternative PI | Prof Judy Bradley |
| Institute / employer | Queen’s University Belfast |
| Project Title | CABIN – (CArdiac Brief INtervention): a feasibility study to promote engagement with Cardiac Rehabilitation through an early, personalised and holistic intervention. |
| length of project | 12 months |
| grant awarded | £58,500.75 |

Dear Professor Fitzsimons

**Results of NICHS research SRC grant process**

Thank you for applying to our research grants programme 2021. Thank you also for your patience whilst we arranged peer review, review by our Scientific Research Committee (SRC), and awaited decision from our Governance Board. We appreciate that it was a long wait.

I am pleased to inform you that your project has been recommended for a grant by our SRC and NICHS Board. So, firstly, congratulations.

The next step is to make any changes / provide requested clarifications to your application that the SRC may have requested, and then formally agree contract and start dates. I have enclosed the following documentation for your approval:

- SRC application feedback, with requested changes / clarifications, where relevant.
- NICHS Research Grants Terms and Conditions,
- NICHS IP & Revenue Sharing Terms, and
- NICHS ResearchFish Policy.
- A NICHS Start Certificate

Please read, sign, and return the attached documentation, as outlined (further details are provided overleaf). Your project cannot commence until all relevant paperwork (including any relating to governance / ethical approval, where relevant) is signed and returned to us.

If you have any queries please do not hesitate to contact our Research and Impact Manager, details overleaf. In the meantime, I wish you all the very best with your research.

Yours sincerely,

Fidelma Carter

Public Health Director

**SRC application feedback^[1]^**

The Committee judged the application to be good-very good value for money (3 out of 4). It was also judged to be a good-very good fit with NICHS’s aims and objectives (2.88 out of 4 on “benefit for NICHS”). Whilst the Committee scored the application as good in terms of scientific quality (2.3 out of 4), they felt that the application did not sufficiently describe the cardiac rehab programme. It wasn’t clear at what time point it would take place, what does it would involve, where it would be done, if it was just advice. It was noted that given that the application was following on from PhD research, there should have been more detail readily available. The Committee would have liked the research team to have included an examination of why people do not attend. Overall score: 2.8 out of 4.

**Action:** clarify / describe the CR programme involved in the study in a little more detail.

**Contract agreement**

Please sign and return this letter to indicate your agreement to the contract terms and conditions, within **three weeks** of letter date. An electronic copy will be accepted in the first instance, but note a hard (posted) copy is required.

**Start certificate**

Please sign and return your start certificate within **three months** of the letter date. An electronic copy will be accepted in the first instance, but note a hard (posted) copy is required.

Signatures are required, **on both documents**, from:

- the grant holder (principal applicant) and
- an authorised representative from the Research Office of the respective institution.

Digital and/or scanned images of signatures are accepted.

Principal Applicant signature is also taken to be agreement on behalf of the named alternative PI. Please return all documents to the Research & Impact Manager (details at end of the letter).

**Ethical approval / governance**

A Grant may not commence until all necessary ethical committee approvals have been obtained. A copy of these must be forwarded to NICHS prior to commencement unless included in the application.

**Start dates**

Your project is expected to start (unless there are exceptional circumstances) within **6 months** of the date of the grant offer letter. You require permission to extend or delay a start date, and this must be requested formally, (i.e. in writing), and within 1 month of this letter date**.** Failure to do so will result in the automatic withdrawal of the grant offer.

**Reporting on progress and promotional activity**

Throughout the duration of your grant, you will be required to submit research evaluation data on an annual basis, status updates throughout the year, and other updates to support fundraising and promotional activity as detailed in Sections 6 and 8 of the Research Grants Terms and Conditions.

**Changes to your proposed work**

During the period of your grant, any changes to the project, in whatever respect, must be agreed in advance with NICHS, and requested in a signed formal letter. All proposed changes must be directed to the Research & Impact Manager at the earliest opportunity. Significant and/or unwarranted deviations from your agreed project may result in contract termination.

**Claiming payments**

Grant money should be reclaimed retrospectively by your Accounts Department 3 months in arrears; please refer to Section 4 of the Terms and Conditions for additional information. Please quote your reference number, on all correspondence. To assist in our record keeping and processing of accounts, we request that you forward the details of the university finance contact who will manage your account, and university code for your project, as soon as possible.

| **Please tick to indicate that you have received, read and acted on the following documents:** | |
| --- | --- |
| cover letter (including SRC feedback) | ☒ |
| NICHS Start Certificate | ☒ |
| NICHS Research Grants Terms and Conditions | ☒ |
| NICHS IP & Revenue Sharing Terms | ☒ |
| NICHS ResearchFish Policy | ☒ |

| **Declaration (please sign)** | | | |  | |
| --- | --- | --- | --- | --- | --- |
|  | | | |  | |
| We, the undersigned, accept and agree to abide by the NICHS Research Grants Terms and Conditions as stated in the Award Letter / associated documents. | | | | | |
|  | | | | | |
| **Principal Applicant/ Grant holder** | |  | | | |
|  |  |  | | | |
| Name | Professor Donna Fitzsimons |  | | | |
| Signature |  | date | 29 May 2024 | |  |
|  |  |  | | | |
| **Authorised representative from the Host Institution** | |  | | | |
|  |  |  | | | |
| Name | Cheryl McInnes |  | | | |
| Signature |  | date | 29 May 2024 | |  |
|  |  |  |  | |  |

Please return all documentation to the Research & Impact Manager:

[radmin@nichs.org.uk](mailto:radmin@nichs.org.uk)

NICHS, 21 Dublin Road, Belfast, BT2 7HB

1. you must make any changes / clarifications requested by SRC, and return to NICHS, before your grant can become active.
